# Supplementary material for: Critical Care Cycling Study (CYCLIST) trial protocol: a randomised controlled trial of usual care plus additional in-bed cycling sessions versus usual care in the critically ill
Source: BMJ Open. 2017 Oct 22;7(10):e017393. doi: 10.1136/bmjopen-2017-017393 (PMC5665265; doi:10.1136/bmjopen-2017-017393)
Supplement: Supplementary file 1 [file bmjopen-2017-017393supp001.pdf]

Substitute Decision Maker Information Sheet

CYCLIST

Project Title	**Critical Care In-bed CYCLIng Study: A Preliminarily Randomised Controlled Trial: CYCLIST**
Site	Princess Alexandra Hospital
Principal investigator	Mr. Marc Nickels
Contact Person	Mr. Marc Nickels (07) 3176 2401,<br>or Mrs. Chelsea Davis (07) 3176-5523
Address	Physiotherapy Department, Princess Alexandra Hospital, Ipswich Road, Woolloongabba, QLD 4102
Phone Number	07 3176 2401 or (07) 3176-5523

Participant Information Sheet

Participation in this research is voluntary. Acting on behalf of the patient, we kindly ask that you read this information sheet about this clinical trial. If you are satisfied with the explanation and agree for the patient to participate in this trial, please provide your consent on the form provided. You should keep a copy of this sheet for your and for the future reference of the patient for who you are acting on behalf of.

Introduction

This study aims to investigate whether in-bed cycling in addition to standard care reduces the rate of thigh skeletal muscle wasting and is associated with improved functional and cognitive outcomes, in critically ill patients requiring more than 48 hours of mechanical ventilation. The study will also determine if patients who participate in in-bed cycling sessions are able to walk better after discharge from intensive care compared to people who receive standard care alone. This study will also investigate which factors are linked to better outcomes.
Patients who participate in the study will be randomly allocated into either an intervention group that receives in-bed cycling sessions in addition to standard care, or into a control group that receives standard care.

Background to experiment

Current physiotherapy exercise interventions with patients in intensive care do not occur until a patient is reliably able to follow instructions. Research has shown that patients rapidly lose muscle mass whilst they are in ICU. Recent studies have also shown that cycling in-bed whilst in ICU is safe. Research has also shown that patients who complete in-bed cycling whilst in ICU have improved ability to walk at hospital discharge. Currently it is not known what the effect of in-bed cycling is on maintaining thigh muscle mass. Ultrasound is a safe way to assess patients muscle mass without any radiation exposure that occurs with other investigations such as x-rays and computerised tomography scans.

Description of Experiment - methods and demands

This study will test if in-bed cycling in addition to standard care affects thigh muscle loss and if there is any difference in patients' time to walk and distance walked one week after intensive care discharge.

If you chose for the patient for whom you are the substitute decision maker for to participate this means:

a) The patient will be allocated, by a random (chance) selection process, to one of the following groups:
   - Standard care (control group): Physiotherapy exercise interventions that includes sitting on the edge of the bed, moving from a bed to a chair and walking.
   - Cycling group (in-bed cycling intervention group): at least five 30 minute in-bed cycling sessions in addition to 'standard care'. In-bed cycling sessions will continue until the patient is discharged from ICU and has completed a minimum of 5 in-bed cycling sessions. The in-bed cycling sessions will continue in the acute hospital ward if the patient is discharged from ICU prior to completing 5 in-bed cycling sessions).
b) Sonographers will use ultrasound to measure the size of the patients' thigh muscles during their stay in intensive care and one week after they leave the intensive care unit.
c) Physiotherapists will measure the patients' arm, leg muscle and hand grip strength.
d) A clinical nurse will record any incidence of confusion whilst the patient is in ICU.
e) Information about the time taken for the patient to commence standing, sitting out of bed, and walking both without assistance will be recorded.
f) One week after discharge from intensive care a physiotherapist will measure how far the patient can walk in 6 minutes.
g) Information about the patient such as; gender and age, and the patients' medical condition including; diagnosis, hospital length of stay, nutritional status, that is recorded in your hospital record will be accessed by researchers to aid in result analysis.
h) You will be asked questions about your quality of life in hospital and after you return home from hospital. You may also be asked questions about your experiences with treatments you received in hospital.
i) Data collected during this study may be utilised for future research purposes following appropriate subsequent ethical review and approval.

Risk & Discomfort

Other research has established that in-bed cycling is a safe exercise for critically ill patients to participate in. It is possible that in-bed cycling may increase pain or discomfort. However, care will be taken to minimise any potential discomfort that may be experienced, and pain relief medication may be increased if deemed appropriate by the doctors. Patients will be able to request for the in-bed cycling session to be stopped.

Benefits

There may be some benefit but we don't know how much direct benefit there will be to patients participating in the study. It is possible that participation in this study will reduce patients' amount of thigh muscle wasting due to lack of use. This may correspond to a patients' improved ability to walk following a period of critical illness. We also think that participation will benefit patients, and hospitals in the future, and you and the patient may feel satisfaction at your contribution to improving health care through research.

Withdrawing from the Study

Participation is entirely voluntary and if you decide the patient will not participate in this study this will not affect the medical care or treatment of the patient (for whom you are substitute decision maker),

in any way. If you choose for the patient to participate, you are free to withdraw your consent and to discontinue participation of the patient at any time, by telling the research nurse. Choosing for the patient not to participate or withdrawing your consent for their participation will not affect the treatment of the patient (for whom you are the substitute decision maker) for in any way.

Confidentiality

Data collected during this study will be treated confidentially. The research nurse and assistants will store data about the patient using a unique research number. The information will be safely stored at the hospital and Queensland University of Technology. Combined patient results of this study will be published in scientific journals and presented at conferences. However, the patient will not be referred to by name and your personal identity will not be revealed in any publication or report, without specific prior approval. Research data may be accessed by auditors, the ethics committee or regulatory authorities. All research records will be confidentially destroyed 7 years after the study.
Data collected during this study may be utilised for future research purposes following appropriate subsequent ethical review and approval. If data is utilised for future research purposes, it will continue to be treated confidentially.

Contact

If you have any questions now, or at a later time, we hope and expect that you will ask us. Please contact any of the researchers named on this form by contacting *the hospital principal investigator or research nurse*, and we will be happy to answer your questions. Contact details are at the top of this form.

Metro South HHS Human Research Ethics Committee (HREC), (EC00167) and the Queensland University of Technology HREC (EC00171) have approved this study. Should you wish to discuss the study with someone not directly involved, in particular, any matters concerning policies, information about the conduct of the study or the rights of the participant, or if you wish to make a confidential complaint at any time, you may contact;

Coordinator of the Metro South HHS Human Research Ethics Committee,
Translation Research Institute, Level 7,
Woolloongabba QLD 4102
Telephone (07) 3443-8049,
email: Ethicsresearch.pah@health.qld.gov.au

Participant Consent Form

HREC No:	HREC/16/QPAH/193
Project Title:	Critical Care In-bed CYCLIng STudy Preliminary Randomised Controlled Trial: CYCLIST
Name of Researchers:	Mr Marc Nickels, Senior Physiotherapist, Princess Alexandra Hospital, Tel: (07) 3176-2401; Email: marc.nickels@health.qld.gov.au
	Dr Steven McPhail, Princial Research Fellow, Centre for Functioning and Health Research, Tel: (07) 3406 2266; Email: steven.mcphail@health.qld.gov.au

Thank you for agreeing for the patient for whom you are a substitute decision maker to participate in this important research study. Although you or they may not benefit personally, you will help provide valuable information to help us to deliver safe and effective care.

I have had the contents of this information sheet explained to me and I have been provided with a copy. I agree for the patient for whom I am a substitute decision maker for to be enrolled in the project and understand that they will be randomly allocated to either a usual care group or to a usual care plus in-bed cycling group.

Please read the following carefully, and sign below if you agree with these statements and are happy to for the patient for whom you are acting as a substitute decision maker for to participate in the study:

1. I have read and understood the information sheet and this consent form.
2. I have had the opportunity to ask questions about the study and these have been answered to my satisfaction.
3. I understand that this project is for research and that participation may not have a direct benefit to the participant.
4. I have been informed that the information collected about the participant in this study will remain confidential and will be adequately safeguarded, and that when results are published, they will be presented in such a way that individuals cannot be identified.
5. I understand that if I do agree for the patient to participate, I and they are free to withdraw our consent and to discontinue participation at any time without comment, and with no effect on their treatment or relations with the Hospital in any way, but that I do need to tell the research staff if I wish to withdraw the patient for whom I am a substitute decision maker for.
6. If I have any questions or comments about the study at any time I am free to contact Mr Marc Nickels on (07) 3176-2401 or the research nurse on (07) 3176 5523.
7. If I have any complaints about the ethical conduct of the study, I may direct these to the, Coordinator of the Ethics Committee, Princess Alexandra Hospital, on (07) 3176-8049.

I agree for the patient for whom I am a substitute decision maker for, to participate in the study and I give permission for authorised study personnel to extract details that pertain to this study from the patients' hospital medical record.

Name: ……………………………………………Signature………………………….. Date: __/__/__

Witness: .... Signature………………………….. Date: __/__/__

Enrolled by: .... Signature………………………….. Date: __/__/__

Revocation of Consent Form - Participant

HREC No:	HREC/16/QPAH/193
Project Title:	Critical Care In-bed CYCLIng STudy Preliminary Randomised Controlled Trial: CYCLIST
Name of Researchers:	Mr Marc Nickels, Senior Physiotherapist, Princess Alexandra Hospital, Tel: (07) 3176-2401; Email: marc.nickels@health.qld.gov.au
	Dr Steven McPhail, Princial Research Fellow, Centre for Functioning and Health Research, Tel: (07) 3406 2266; Email: steven.mcphail@health.qld.gov.au

(TO BE USED FOR PARTICIPANTS WHO WISH TO WITHDRAW FROM THE PROJECT)

- I hereby wish to WITHDRAW my consent for the patient for whom I am a substitute decision maker for to participate in the research proposal described above.
- I understand that such withdrawal WILL NOT jeopardise any treatment of the patient for whom I am a substitute decision maker for or relationship with the Princess Alexandra Hospital or Queensland University of Technology.

Participant's Name (printed)

____

______________________________________ ______________________

Signature Date

Please send to:
Clinical Research Nurse
Intensive Care Unit
Princess Alexandra Hospital
199 Ipswich Rd
Woolloongabba QLD 4102
