# Supplementary material for: Critical Care Cycling Study (CYCLIST) trial protocol: a randomised controlled trial of usual care plus additional in-bed cycling sessions versus usual care in the critically ill
Source: BMJ Open. 2017 Oct 22;7(10):e017393. doi: 10.1136/bmjopen-2017-017393 (PMC5665265; doi:10.1136/bmjopen-2017-017393)
Supplement: Author's manuscript [file bmjopen-2017-017393.draft_revisions.pdf]

BMJ Open

BMJ Open is committed to open peer review. As part of this commitment we make the peer review history of every article we publish publicly available.

When an article is published we post the peer reviewers' comments and the authors' responses online. We also post the versions of the paper that were used during peer review. These are the versions that the peer review comments apply to.

The versions of the paper that follow are the versions that were submitted during the peer review process. They are not the versions of record or the final published versions. They should not be cited or distributed as the published version of this manuscript.

BMJ Open is an open access journal and the full, final, typeset and author-corrected version of record of the manuscript is available on our site with no access controls, subscription charges or pay-per-view fees (http://bmjopen.bmj.com).

If you have any questions on BMJ Open's open peer review process please email editorial.bmjopen@bmj.com

Critical Care Cycling Study (CYCLIST) Trial Protocol: a randomised controlled trial of usual care versus usual care plus additional in-bed cycling sessions in the critically ill

Journal:	*BMJ Open*
Manuscript ID	bmjopen-2017-017393
Article Type:	Protocol
Date Submitted by the Author:	20-Apr-2017
Complete List of Authors:	Nickels, Marc; Princess Alexandra Hospital, Physiotherapy Department<br>Aitken, Leanne M.; City, University of London, School of Health Sciences; Griffith University Menzies Health Institute Queensland, National Centre of Research Excellence in Nursing Interventions for Hospitalised Patients<br>Walsham, James; Princess Alexandra Hospital, Intensive Care Unit; University of Queensland<br>Barnett, Adrian; Queensland University of Technology, Institute of Health and Biomedical Innovation<br>McPhail, Steven; Queensland University of Technology, Institute of Health and Biomedical Innovation; Metro South Hospital and Health Service, Centre for Functioning and Health Research
**Primary Subject Heading**:	Intensive care
Secondary Subject Heading:	Rehabilitation medicine
Keywords:	INTENSIVE & CRITICAL CARE, REHABILITATION MEDICINE, cycle ergometry, muscle atrophy, physiotherapy, critical illness

SCHOLARONE™ Manuscripts

For peer review only

Critical Care Cycling Study (CYCLIST) Trial Protocol: a randomised controlled trial of usual care versus usual care plus additional in-bed cycling sessions in the critically ill

Authors

1. Marc R Nickels [a, *]
2. Leanne M Aitken [b, c, d]
3. James Walsham [d, e]
4. Adrian G Barnett [f]
5. Steven M McPhail [f, g]

a) Intensive Care Unit and Physiotherapy Department, Princess Alexandra Hospital, Brisbane, Australia

b) School of Health Sciences, City, University of London, London, United Kingdom

c) National Centre of Research Excellence in Nursing Interventions for Hospitalised Patients, Menzies Health Institute Queensland, Griffith University, Brisbane, Australia

d) Intensive Care Unit, Princess Alexandra Hospital, Brisbane, Australia

e) University of Queensland, Brisbane, Australia

f) School of Public Health & Social Work and Institute of Health and Biomedical Innovation, Queensland University of Technology, Brisbane, Australia

g) Centre for Functioning and Health Research, Metro South, Brisbane, Australia

* Corresponding author at: Intensive Care Unit and Physiotherapy Department, Princess Alexandra Hospital, Brisbane, Australia, 4102. Tel.: +61 7 3176 2401. E-mail address: marc.nickels@health.qld.gov.au (M. Nickels).

Keywords:

Critical illness; Cycle ergometry; Intensive care units; Muscle weakness; Rehabilitation

For peer review only

ABSTRACT

Introduction

In-bed cycling with critically ill patients has been shown to be safe, feasible and improve physical function outcomes at hospital discharge. The effect of early in-bed cycling on reducing the rate of skeletal muscle atrophy, and associations with physical and cognitive function are unknown.

Methods and analysis

A single centre randomised controlled trial in a mixed medical-surgical intensive care unit (ICU) will be conducted. Adult patients (n= 68) who are expected to be mechanically ventilated for more than 48 hours and remain in ICU for a further 48 hours from recruitment will be randomly allocated into either (1) a usual care group or (2) a group that receives usual care and additional in-bed cycling sessions. The primary outcome is change in rectus femoris cross-sectional area at day 10 in comparison to baseline measured by blinded assessors. Secondary outcome measures include muscle strength, incidence of ICU acquired weakness, handgrip strength, time to achieve functional milestones (sitting out of bed, walking), Functional Status Score in ICU, ICU mobility scale, six-minute walk test one week post ICU discharge, incidence of delirium and quality of life (EQ-5D-5L). Quality of life assessments will be conducted at day 10 post ICU admission and 3 and 6 months post hospital discharge. Participants in the intervention group will complete an acceptability of intervention questionnaire.

Ethics and dissemenimation

Appropriate ethical approval from Metro South Health Human Research Ethics Committee has been attained. Results will be published in peer-reviewed publications and presented at scientific conferences to assist planning of future multi-centre randomised controlled trials (if indicated) that will test in-bed cycling as an intervention to improve the physical, cognitive and health-related quality of life outcomes of critically ill patients.

Trial Registration

This trial has been prospectively registered on the Australian and New Zealand Clinical Trial Registry (ACTRN12616000948493).

For peer review only

Strengths and Limitiations of this study

- The randomised trial design with blinded assessments of skeletal muscle size, strength and function to provide objective measures of difference
- The inclusion of an acceptability questionnaire will provide useful insights for subsequent implementation (if indicated)
- The study may not be powered for all secondary outcomes, and evidence of effect size from pilot data is not available for those measures

For peer review only

Background and rationale

Critically ill patients often require mechanical ventilation for periods greater than 48 hours. It has been identified that skeletal muscle wasting occurs early and rapidly during the first week of critical illness [1]. Despite international recommendations for critically ill patients to commence activity as early as possible [2] it has been identified that exercise interventions are rarely initiated when a patient is on mechanical ventilation [3]. This leads to prolonged immobility and may contribute to the development of intensive care unit acquired weakness (ICUAW) [4]. In-bed cycling using a cycle ergometer has been proposed as a safe and feasible method of introducing early exercise for critically ill patients on mechanical ventilation who are sedated and immobile, this includes patients requiring inotropic support [5-7]. In-bed cycling may also assist in the preservation of muscle architecture. To date only one randomised controlled trial (RCT) utilising in-bed cycling in the critically ill population has been published [8]. This single centre RCT (n = 90) conducted in Belgium, found in-bed cycling to be safe and to improve critically ill patients' 6-minute walk distance, quadriceps force and Short Form-36 (SF-36) physical function scores on hospital discharge [8]. A pilot case-matched study of in-bed Functional Electrical Stimulated (FES) cycling intervention in addition to usual care, found positive physical outcomes observed among the cycling group included less time required to achieve functional milestones, time to stand and time to ambulate independently [9]. In addition, a shorter duration of delirium among those who participated in the Functional Electrical Stimulation in-bed cycling intervention was observed [9].

A recent clinical trial has demonstrated that critically ill patients may experience persistent weakness despite participating in intensive exercise programs whilst they are critically ill [10]. It has been suggested that intensive exercise programs may not be effective if the commencement of these programs is delayed [11]. Early exercise commencement is intended to assist in the maintenance of muscle mass. This may be achieved through a moderation of the inflammatory process [11]. Consequently, the effectiveness of exercise interventions that can commence early during critical illness are necessary to demonstrate if patient outcomes are improved by the early commencement of exercise during a period of critical illness.

Early clinical studies in the field have demonstrated potential for in-bed cycling interventions (with and without Functional Electrical Simulation) to improve physical and cognitive function among critically ill patients. There are currently no published RCTs investigating the effectiveness of in-bed cycling in critically ill patients requiring prolonged mechanical ventilation on quadriceps structure, ICU-acquired weakness and cognitive outcomes. Consequently, further investigation of the effect of in-bed cycling on muscle structure, physical function and cognitive function is warranted.

For peer review only

Objectives

The objectives of this study are to:

(1) Examine whether in-bed cycling in addition to usual care is effective in reducing the rate of rectus femoris cross-sectional area (CSA) atrophy and ICUAW in patients requiring more than 48 hours of mechanical ventilation compared with usual care.

(2) Investigate if in-bed cycling in addition to usual care is associated with better functional and cognitive outcomes in patients predicted to require more than 48 hours of mechanical ventilation compared to usual care.

METHODS

Study Design and Setting

This trial will be a two arm, parallel randomised controlled trial with individual participant allocation and blinding of the primary outcome assessor. It will be conducted in a 25-bed tertiary mixed medical and surgical adult intensive care unit in Brisbane, Australia. Participants will be allocated 1:1 to receive either usual care or in-bed cycling in addition to usual care (Figure 1). In designing this study, the SPIRIT 2013 Checklist was utilised to ensure that all recommended items in a clinical trial were addressed [12].

Consent

It is anticipated that most patients who are eligible to participate in this study in the ICU setting will not be able to provide informed consent at the time of study enrolment. For those patients that may have the capacity to provide informed consent, the Richmond Agitation and Sedation Scale (RASS) will be used to determine if a patient is rated 'Alert and Calm'. If a patient is rated as 'Alert and Calm' on the RASS, the Confusion Assessment Method for the ICU (CAM-ICU) will be used to determine if a patient has had delirium within the preceding 24 hours. Provided a patient passes the RASS and CAM-ICU assessments the treating clinical team will be approached to determine if the patient has the capacity to provide informed consent. Patients without delirium for the preceding 24 hours and deemed to have capacity will be approached to provide their own written informed consent for study participation. For eligible patients considered unable to provide informed consent at the time of study enrolment, substitute decision makers (family members or next of kin), will be approached for written informed consent. The Queensland Civil and Administrative Tribunal (QCAT) have approved an application to provide consent for individuals who are unable to give consent and do not have a next of kin that is accessible to request consent on the patients' behalf. Delayed consent from the patient will be sought once they can provide consent for themselves if they are enrolled using QCAT approval. Participation in the study is voluntary. Patients or their substitute decision

For peer review only

makers are able to withdraw at any time without any negative consequences on the care they would receive, their ongoing relationship with the hospital, or staff involved in their care.

Randomisation

Patients will be individually randomised in a 1:1 ratio to either intervention or usual care group. Blocking (random block sizes) will be used to help balance the groups. An investigator not involved in the screening, consenting, allocation or assessment processes will use computerised random number generation to create the randomisation sequence. A randomisation sequence will be uploaded onto the Research Electronic Data Capture (REDCap) secure web based computer application [13]. The REDCap randomisation module will reveal the group allocation of each patient to the intervention coordinator after a patients' baseline data has been collected.

Participants

The study aims to recruit 68 participants. Adult patients expected to require at least 48 hours of mechanical ventilation will be recruited, with the inclusion and exclusion criteria listed in Table 1. Criteria to guide when to discontinue or not deliver an intervention are listed in Table 2.

Table 1 Inclusion and exclusion criteria

Inclusion	Exclusion
Expected to require more than 48 hours of mechanical ventilation	Pre-existing condition that is likely to impair mobility/ mobility assessment (e.g. significant neurological, musculoskeletal, cognitive or mental health disorder)
Able to provide consent or have a family member consent on their behalf	Neuromuscular disorder or acute primary brain lesion (e.g. traumatic brain injury, intracranial haemorrhage, stroke, or hypoxic brain injury)
Enrolled into the study within 96 hours of ICU admission	Injuries precluding cycle ergometry (e.g. spinal / pelvic / lower limb orthopaedic injuries / open abdominal wound)
Expected to remain in ICU for more than 48 hours following study enrolment	Obesity > 135 kilograms (MOTOmed Letto 2 maximum rated weight capacity)
	Uncontrolled seizures or status epilepticus
	Dire prognosis (e.g. unlikely to survive the current admission)
	Pregnancy
	Children and/or young people (i.e. < 18 years)

Study Intervention

All study patients will receive usual physiotherapy interventions whilst in intensive care. Physiotherapy interventions will include (but are not limited to): respiratory physiotherapy, physical rehabilitation exercise interventions including sitting on the edge of the bed, sit to stand transfers, sitting out of bed and walking. Safety guidelines (see Table 2) will be used to determine if the intervention group patients are able to complete an additional daily 30-minute progressive lower

For peer review only

limb in-bed cycling using a bedside cycle ergometer (MOTOmed Letto 2). During the in-bed cycling exercise interventions the patients' vital signs will be monitored. If the intervention group are in a state of low arousal or sedated they will cycle continuously and passively at a cadence of 20 revolutions per minute. Once the patient can cycle actively the resistance applied by the cycle ergometer will be adjusted to facilitate optimal patient intensity throughout the session (within the specified safety guidelines). This will enable the patient to cycle in-bed either passively or actively with assistance from the cycle ergometer. When the participant is following commands the clinician will verbally encourage the patient to complete in-bed cycling sessions for a duration of 30 minutes. The in-bed cycling sessions will continue until the patient completes a minimum of 5 in-bed cycling sessions, unless the patient is discharged from hospital prior to completing 5 sessions. The intervention will continue in the acute hospital ward if the patient is discharged from ICU prior to completing 5 in-bed cycling sessions. Whilst the patient remains in ICU, the in-bed cycling sessions will continue, up to 28 days' post ICU admission. Patients randomised to the usual care arm do not routinely complete in-bed cycling sessions during their hospitalisation. Any deviations from the planned protocol will be recorded to enable appropriate intervention description and if indicated a per-protocol analysis (in addition to the primary intention to treat analysis).

Participants will not be coerced to complete any intervention or outcome measure. Participants' may be discharged home from the participating acute hospital before they have completed outcome measures at each assessment time-point. If this occurs, participants will be asked to return to the hospital to enable the remaining outcome measures to be completed and expenses related to taxi or parking costs will be reimbursed.

Table 2 Safety guidelines

**Active or passive exercise should not be delivered if:**
Clinician opinion that patient condition unstable
Resting HR < 40 or > 120 or new arrhythmia
Evidence of coronary ischaemia e.g. chest pain or ECG changes
MAP < 60 or SBP > 200 mmHg
SpO_2 < 90%
RASS ≥ 2
Wounds of leg, pelvis or lumbar spine precluding cycle ergometry
Evidence of active bleeding or coagulation disorder: INR >1.8, PLT <50,000/microL. *
Femoral vascular access ** e.g. dialysis catheter, IABP, ECMO or lower limb arterial line ***
Acute DVT or PE
**Active exercise should not be delivered if:**
> 20 µg/min of noradrenaline or comparable inotropic or vasopressor support
FiO_2 > 0.55 or PEEP > 10 cmH_2O
RR > 30 with adequate ventilatory support
Temperature > 39° Celsius
**Stopping criteria: active or passive exercise should cease if:**

For peer review only

HR < 50 or > 140, or new arrhythmia develops (including ventricular ectopic or new-onset AF)
Evidence of coronary ischaemia e.g. chest pain or ECG changes
MAP < 60 mmHg
SBP > 200 mmHg
Clinical signs of cardiorespiratory distress
SpO2 < 90% for more than 1 minute
Patient request to stop therapy

HR, heart rate; ECG, electrocardiogram; FIO2, fraction of inspired oxygen; MAP, mean arterial pressure; SBP, systolic blood pressure; DBP, diastolic blood pressure; SpO2, saturation of peripheral oxygen; RASS, Richmond Agitation Sedation Score; INR, international normalised ratio; PLT, platelets; microL, microlitre; IABP, intra-aortic balloon pump; ECMO, extracorporeal membrane oxygenation; DVT, deep vein thrombosis; PE, pulmonary embolism; PEEP, positive end expiratory pressure; RR, respiratory rate; AF, atrial fibrillation

** Values outside this range would be tolerated if patient therapeutically anticoagulated*

*** Other than femoral central line*

****If a femoral vascular access is inserted unilaterally the contralateral leg may be cycled unilaterally.*

Outcomes

Table 3 provides a summary of the outcome measures. The primary outcome is the percentage of change in rectus femoris CSA measured at baseline (within 24 hours of study enrolment) and day 10 (post study enrolment), measured by a blinded assessor. Secondary outcome measures in the CYCLIST study are muscle strength, physical function, cognition, quality of life, and acceptability of intervention.

Table 3 Descriptions of outcome measures for CYCLIST RCT

Assessment Component	Outcome Measure	Description
Muscle Morophology	Ultrasound	RF CSA, AP thickness of RF and VI. Measured in triplicate on right anterior thigh one third distance from superior patella to ASIS Patient positioned in supine, thirty degress head elevation [14]
Muscle Strength	MRC Sum Score	Standardised sum of twelve MMTs, three MMTs per limb Score ≤ 48 indicative of ICU acquired weakness [15]
	Handgrip strength dynamometry	Triplicate bilateral measurement using a Jamar Digital Dynamometer (Lafayette) with seated patient [16]
Physical Function	ICU Mobility Scale	Best level of function achieved in ICU using an eleven-point ordinal scale [17]
	FSS-ICU	Patients' function measured an eight-point ordinal scale [18 19]
	Functional milestones	Time to achieve functional milestones: Sit out of bed, time to stand, mobilise with assistance and mobilise independently
	6-minute walk test	Sub-maximal endurance test of distance walked by a patient in six minutes [20]
Cognition	CAM-ICU	Incidence and recorded episodes of acute delirium [21]
Quality of Life	EQ-5D-5L	EuroQol five dimensions questionnaire five level scale [22]
Intervention Acceptability	Customised questionnaires	Questionnaire about the acceptability of the in-bed cycling intervention

For peer review only

RF, rectus femoris; CSA, cross sectional area; VI, vastus intermedius; AP, anterior posterior; ASIS, anterior superior illiac crest; MRC, Medical Research Council; MMT, manual muscle test; ICU, intensive care unit; FSS-ICU, Funcitonal Status Score for the Intensive Care Unit; CAM-ICU Confusion Assessment Measure for the Intensive Care Unit; EQ-5D-5L, EuroQol five dimensions questionnaire five level scale.

Demographic information such as age, gender and diagnostic code will be collected. Illness related information including length of mechanical ventilation, ICU (LOS) and hospital LOS and discharge destination, illness severity (APACHE II [23] and APACHE III [24], Sequential Organ Failure Assessment (SOFA) [25] score, pre-morbid co-morbidities, neuromuscular blockade and sedation medications administered, nutrition received, cumulative fluid balance, patient height and weight, and body mass index will also be collected.

Sample Size

Based on the repeated measures design this study will have 80% power to detect a difference of 2.9% on our primary outcome (change in rectus femoris CSA) between the intervention and usual care groups, (assuming type I error 0.05, standard deviation of 6%, and within-patient correlation of 0.5 between assessments, and after accounting for an up to 20% drop out rate). A total of 68 patients (34 in each group) will be recruited into the study. A previous study of acute muscle wasting in critically ill patients [1] reported the mean change in rectus femoris CSA was –17.7% with a 95% confidence interval of –20.9% to –4.8%.

Data Collection

The study data will be collected at the time points summarised in Table 4.

Table 4 CYCLIST summary of timepoints of assessments

	Baseline	Day 3	Day 7	Day 10	ICU discharge	1 week post ICU discharge*	3, 6 months' post hospital discharge
Severity of illness							
APACHE II and III	✓						
SOFA	✓	✓	✓	✓	✓		
Muscle Morphology							
Quadriceps Ultrasonography	✓	✓	✓	✓		✓	
Strength Measures							
MRC Sum Score					✓	✓	
Handgrip dynamometry					✓	✓	
Physical Function Measures							
ICU Mobility Scale					✓		
FSS-ICU					✓		
Functional Milestones	✓	✓	✓	✓	✓	✓	
6-MWT						✓	

Cognition							
CAM-ICU	✓	✓	✓	✓	✓		
Quality of Life							
EQ-5D-5L				✓			✓
Acceptability of Intervention							
					✓**		
Patient Outcomes							
ICU Length of Stay					✓		
Hospital Length of Stay						✓***	
Acute discharge destination						✓***	
Mortality					✓	✓***	✓

ICU, intensive care unit; APACHE, Acute Physiology and Chronic Health Evaluation; SOFA, Sequential Organ Failure Assessment score; MRC, Medical Research Council; FSS-ICU, functional status score in the intensive care unit; CAM-ICU, confusion assessment measure in intensive care; EQ-5D-5L, EuroQol five dimensions. questionnaire five level scale.

** 1 week post ICU discharge or at acute hospital discharge if sooner.*

*** At completion of in-bed cycle ergometry sessions*

**** Measured at acute hospital discharge*

The management of study data will be dependent on the type of data collected. Baseline data will be entered directly into Research Electronic Data Capture (REDCap) designed digital clinical trial workflow management software [13]. Ultrasound results will be uploaded onto the secure hospital based AGFA IMPAX 6.5.3.1005 Medical Image Viewer application. In-bed cycling session data, physical assessment measures, acceptability of in-bed cycling intervention and quality of life questionnaires will be recorded initially onto research data sheets. All information recorded on data sheets will be subsequently entered into the REDCap application.

Assessments of muscle size, strength and function will be performed by sonography and physiotherapy assessors blinded to patient treatment group. Sonographers will be responsible for analysing and scoring all ultrasound images. Physiotherapy assessors will assess patients' muscle strength and function at ICU discharge and 7-days post ICU discharge. A physiotherapy assessor will also conduct a 6-minute walk test 7-days following ICU discharge.

A physiotherapist not involved in blinded outcome assessment will be responsible for conducting the in-bed cycling sessions, and the remaining 'usual care' physiotherapy will be completed by hospital department physiotherapists not involved in the study. Additionally, the primary statistician will be blinded to treatment group allocation.

For peer review only

To minimise the chances of unintentional unblinding, at the beginning of each assessment the blinded assessors will clearly state that they wish to complete the assessment process without knowing which group the patient was allocated too. If any blinded assessors were to become unblinded to group allocation for any patient (e.g., through inadvertent revelation by a patient being assessed or unanticipated exposure to a patient taking part in an in-bed cycling session), they have been instructed to report this to the intervention coordinator (MRN) who will also record when this occurred.

ANALYSIS

Data will be analysed and reported using intention to treat principles (primary analysis). A per protocol analysis will be conducted to assist in determining the efficacy of the in-bed cycling protocol if variation from the planned protocol occurs for a substantial proportion of patients. The per protocol analysis will include only patients who adhered to the protocol and received at least 80% of training sessions (minimum of 4 sessions).

Descriptive statistics and generalized linear mixed models will be used to examine the effect of group allocation (intervention vs. usual care) on the primary and secondary outcomes. As this is a randomised trial we do not plan to adjust for potential confounders (e.g., age, gender, comorbidities), but will compare the characteristics of the sample by treatment group and may adjust if a potential confounder differs greatly between groups.

If blinded sonographers are unable to complete an ultrasound measurement at designated time-points, then the primary outcome will not be recorded but a physiotherapist experienced in musculoskeletal ultrasound will measure thigh muscle size to assist in imputation of the missing primary outcome data. Statistical analysis of available data will be used for the primary analysis when data is completely missing. Multiple imputation will be conducted if more than 20% of outcome data is missing. Additional planned secondary analyses are listed in table 5. The principal investigators will determine if study protocol modifications are required and any modifications to the existing protocol will be declared.

Table 5 Planned secondary analyses

Muscle wasting at baseline, day 3, day 7 and 1 week post ICU discharge
Muscle wasting adjusted for number of failing vital organs *
Muscle wasting adjusted for severity of illness on admission to ICU **
Muscle wasting adjusted for the number of days prior to a patient commencing active activity
Muscle wasting adjusted for the patients' cumulative fluid balance on the day of the ultrasound scan

For peer review only

Relationship between muscle wasting and participants' nutritional intake whilst in ICU
Relationship between sedative and paralytic medications and muscle wasting
Cost comparision of hospitalisation for both the intervention and usual care groups ***
Hospital readmissions post acute hospital discharge over a two year time period ****
Mortality *****

Utilising: * Sequential Organ Failure Score (SOFA), ** APACHE II and APACHE III data, *** health service utilisation data, **** Queensland Hospital Admitted Patient Data Collection (QHAPDC), ***** Queensland Health Statistical Services Branch

TRIAL MANAGEMENT

The principal investigator (MRN) will oversee the conduct and progress of the trial. The principal investigator will screen the daily admission to ICU lists and liaise with the treating medical teams to optimise participant enrolment. No interim analyses are planned. The principal investigator will ensure all research personnel are appropriately orientated and trained, oversee recruitment and report to a trial safety monitoring committee who will monitor the progress and conduct of the trial. The trial safety monitoring committee will include: a physiotherapist and researcher experienced in the safe conduct of clinical trials with physiotherapy interventions; the trial coordinator and principal investigator; a critical care nurse who is also experienced in the safe conduct of clinical trials in critical care settings and two ICU medical consultants experienced with the safe conduct of clinical trials in intensive care units. One of the ICU medical consultants is employed externally to the study site. The principal investigator will provide an update report to the Safety Monitoring Committee on a monthly basis (and additional ad-hoc reports if an adverse event was to occur). Additionally any Serious Adverse Events will be reported to the approving Metro South Human Research Ethics Committee that is overseeing the study.

DISSEMINATION

Study results will be disseminated via publication in peer-reviewed literature and scientific conference presentations. It is anticipated that media releases in lay form will be completed to target the general community. Study results will also be placed on a university website for viewing by participants and other interested parties. There are no publication restrictions. Authorship eligibility guidelines as outlined in The Australian Code for the Responsible Conduct of Research [26] and consistent with those proposed by the International Committee of Medical Journal Editors will be followed to determine authorship [27].

For peer review only

DISCUSSION

Survival rates following critical illness are improving [28]; however, patients are experiencing deficits in physical and cognitive function that do not equal age matched peers 5 years after an episode of critical illness [29]. The delayed initiation of rehabilitaitve exercise interventions with critically ill patients may explain the limited effectiveness of clinical trials that have studied with effect of exercise interventions on patients functional outcomes [11]. A binational clinical trial that aimed to commence exercise interventions as early as possible with mechanically ventilated patients reported that despite the presence of a dedicated early mobility team, patient mobilisation out of bed while mechanical ventilation was in-situ was rare [30]. These results are substantiated by point prevalance studies from Australia, New Zealand and Germany that report that in 1281 patient days only one patient with an endotracheal tube was mobilised out-of-bed [3 31]. The presence of an endotracheal tube is negatively correlated with out-of-bed mobilisation in the United States with a reported odds ratio of 0.1, [95% CI, 0.05-0.2] [32]. Studies have reported that following a period of critical illness rehabilitative interventions do not hasten recovery when they are provided post acute hospital discharge [33 34]. Consequently this trial will provide valuable clinical trial evidence regarding the effect of an exercise intervention initiated early in the critically ill patients illness. Specifically, it will report empirical data about the effect of the intervention on the rate of skeletal muscle wasting, and whether early exercise interventions that can be feasibly implemented among people who are mechanically ventilated are associated with improved physical, cogntive and health related quality of life outcomes.

CYCLIST is a randomised controlled trial that is powered to investigate if the early application of an additional in-bed cycling intervention is able to reduce the rate of skeletal muscle atrophy of patients' quadriceps muscle during and immediately following a period of critical illness, in comparison to usual care.

The strengths of this study are the implementation of the in-bed cycling intervention as soon as feasible (including while patients are still mechanically ventilated), with clear commencement and stopping rules. Another strength of the study is the blinded assessment of muscle structure, strength and function assessments. Measurement of the effect of early in-bed cycling on quality of life post hospital discharge is another strength that will assist to demonstrate if potential gains made early during a period of critical illness correspond to lasting functional improvements. It is expected that this study will also provide insights regarding the feasabilty of the in-bed cycling intervention with critically ill patients from the rates of compliance and completion of the in-bed cycling exercise

For peer review only

intervention. The acceptability of in-bed cycling intervention from the perspective of critically ill patients' will be sought through a questionnaire and provide new information to inform potential implementation strategies for this intervention. A limitation of this study is that it is being conducted at a single centre and therefore results may need to be interpreted will caution.

Word Count: 2993

Contributors

MRN contributed to study conception, design, grant acquisition, trial management (including intervention coordination, data collection), data management, protocol drafting, appraisal and editing. SMM, LMA and JW contributed to study conception, design, grant acquisition, analysis plan, data management, protocol drafting, appraisal and editing. AGB contributed to study analysis plan, grant acquisition, data management, protocol drafting, appraisal and editing. All authors read and approved the final manuscript.

Funding

This is an investigator initiated trial without and external sponsors. Following a competitive peer review process Metro South Health Study, Education and Research Trust Account (SERTA) awarded this study a grant in 2015. MRN has also been awarded a competitive Princess Alexandra Research Support Scheme Postgraduate Scholarship to conduct this study. SMM (#1090440) and AGB (#1117784) are supported by National Health and Medical Research Council (NHMRC) fellowships. In addition, this study is receiving in-kind support in the form of personnel and administrative support from Metro South Health (Queensland) to enable the study to be conducted. No funding body had a role in study design, collection, management, analysis, and interpretation of data; writing of the report; and the decision to submit the report for publication.

Competing interests

None declared

Access to data

The principal investigator MRN will have full access to the final trial dataset. Associate investigators SMM, LMA, JW and AGB will have access to the final de-identified trial dataset. Other investigators will be granted access to sections of the final trial dataset. Dataset sections may include de-

identified demographic data and information that pertains to data collected by the professional discipline of the associate investigator.

Ancillary and post-trial care

Given the low risk of adverse events no specific provisions for ancillary and post-trial care have been made. If patients from either allocated group require follow-up services appropriate referrals for follow-up care would be made.

Ethics approval

Research Ethics Approval

Human research ethics approvals for this study have been gained from Metro South Human Research Ethics Committee (EC00167) on the 28th April 2016 (HREC/16/QPAC/193), and subsequent approval following an administrative review from Queensland University of Technology Human Research Ethics Committee (QUT reference number: 1600000441). Ethics approval has been granted until 28th April 2019.

Site specific approval (SSA) has been granted by Metro South Centres for Health Research, Research Governance (SSA/16/QPAH/195) on the 1st June 2016.

CYCLIST Study Protocol Version 2.1 dated 30/03/2017 was approved on 13/04/2017.

Participant recruitment commenced on 26th July 2016.

Provenance and peer review

Not commissioned; externally peer reviewed.

References:

1. Puthucheary ZA, Rawal J, McPhail M, et al. Acute Skeletal Muscle Wasting in Critical Illness. *JAMA* 2013;310(15):1591-600.

2. Gosselink R, Bott J, Johnson M, et al. Physiotherapy for adult patients with critical illness: recommendations of the European Respiratory Society and European Society of Intensive Care Medicine Task Force on Physiotherapy for Critically Ill Patients. *Intensive Care Med* 2008;34(7):1188-99.

3. Berney SC, Harrold M, Webb SA, et al. Intensive care unit mobility practices in Australia and New Zealand: a point prevalence study. *Crit Care Resusc* 2013;15(4):260-5.

4. Fan E. Critical illness neuromyopathy and the role of physical therapy and rehabilitation in critically ill patients. *Respir Care* 2012;57(6):933-44; discussion 44-6.

5. Kho ME, Martin RA, Toonstra AL, et al. Feasibility and safety of in-bed cycling for physical rehabilitation in the intensive care unit. *Journal of critical care* 2015;30(6):1419.e1-19.e5.

6. Kho ME, Molloy AJ, Clarke FJ, et al. TryCYCLE: A Prospective Study of the Safety and Feasibility of Early In-Bed Cycling in Mechanically Ventilated Patients. *PloS one* 2016;11(12):e0167561.

7. Pires-Neto CR, Fogaça Kawaguchi YM, Sayuri Hirota A, et al. Very Early Passive Cycling Exercise in Mechanically Ventilated Critically Ill Patients: Physiological and Safety Aspects - A Case Series. *PloS one* 2013;8(9):e74182.

8. Burtin C, Clerckx B, Robbeets C, et al. Early exercise in critically ill patients enhances short-term functional recovery. *Crit Care Med* 2009;37(9):2499-505.

9. Parry SM, Berney S, Warrillow S, et al. Functional electrical stimulation with cycling in the critically ill: A pilot case-matched control study. *Journal of critical care* 2014;29(4):695.e1-e7.

10. Moss M, Nordon-Craft A, Malone D, et al. A randomized trial of an intensive physical therapy program for acute respiratory failure patients. *American journal of respiratory and critical care medicine* 2016;193.

11. Hodgson CL, Iwashyna TJ, Schweickert WD. All That Work and No Gain: What Should We Do to Restore Physical Function in Our Survivors? *American journal of respiratory and critical care medicine* 2016;193(10):1071.

12. Chan A-W, Tetzlaff JM, Gøtzsche PC, et al. SPIRIT 2013 explanation and elaboration: guidance for protocols of clinical trials. *BMJ (Clinical research ed)* 2013;346(jan08 15):e7586-e86.

13. Harris PA, Taylor R, Thielke R, et al. Research electronic data capture (REDCap)—A metadata-driven methodology and workflow process for providing translational research informatics support. *Journal of Biomedical Informatics* 2009;42(2):377-81.

14. Tillquist M, Kutsogiannis DJ, Wischmeyer PE, et al. Bedside Ultrasound Is a Practical and Reliable Measurement Tool for Assessing Quadriceps Muscle Layer Thickness. *Journal of Parenteral and Enteral Nutrition* 2014;38(7):886-90.

For peer review only

15. De Jonghe B, Sharshar T, Lefaucheur JP, et al. Paresis acquired in the intensive care unit: a prospective multicenter study. *JAMA* 2002;288(22):2859-67.

16. Parry SM, Berney S, Granger CL, et al. A new two-tier strength assessment approach to the diagnosis of weakness in intensive care: an observational study. *Crit Care* 2015;19:52.

17. Hodgson C, Needham D, Haines K, et al. Feasibility and inter-rater reliability of the ICU Mobility Scale. *Heart Lung* 2014;43(1):19-24.

18. Huang M, Chan KS, Zanni JM, et al. Functional Status Score for the ICU: An International Clinimetric Analysis of Validity, Responsiveness, and Minimal Important Difference. *Critical Care Medicine* 2016:1.

19. Ragavan VK, Greenwood KC, Bibi K. The Functional Status Score for the Intensive Care Unit Scale: Is It Reliable in the Intensive Care Unit? Can It Be Used to Determine Discharge Placement? *Journal of Acute Care Physical Therapy* 2016;7(3):93-100.

20. ATS statement: guidelines for the six-minute walk test. *American journal of respiratory and critical care medicine* 2002;166(1):111-7.

21. Ely EW, Margolin R, Francis J, et al. Evaluation of delirium in critically ill patients: validation of the Confusion Assessment Method for the Intensive Care Unit (CAM-ICU). *Crit Care Med* 2001;29(7):1370-9.

22. Herdman M, Gudex C, Lloyd A, et al. Development and preliminary testing of the new five-level version of EQ-5D (EQ-5D-5L). *Quality of Life Research* 2011;20(10):1727-36.

23. Knaus WA, Draper EA, Wagner DP, et al. APACHE II: A severity of disease classification system. *Critical Care Medicine* 1985;13(10):818-29.

24. Knaus WA, Wagner DP, Draper EA, et al. The APACHE III prognostic system. Risk prediction of hospital mortality for critically ill hospitalized adults. *Chest* 1991;100(6):1619-36.

25. Vincent JL, Moreno R, Takala J, et al. The SOFA (Sepsis-related Organ Failure Assessment) score to describe organ dysfunction/failure. On behalf of the Working Group on Sepsis-Related Problems of the European Society of Intensive Care Medicine. *Intensive Care Med* 1996;22(7):707-10.

26. Australian Code for the Responsible Conduct of Research. *Australian Government* 2007:1-41.

27. Editors. ICoMJ. Recommendations for the Conduct, Reporting, Editing and Publication of Scholarly Work in Medical Journals Available from: http://www.ICMJE.org. [accessed 01/27/2017.

28. Iwashyna TJ, Cooke CR, Wunsch H, et al. Population Burden of Long - Term Survivorship After Severe Sepsis in Older Americans. *Journal of the American Geriatrics Society* 2012;60(6):1070-77.

29. Herridge MS, Tansey CM, Matté A, et al. Functional disability 5 years after acute respiratory distress syndrome. *N Engl J Med* 2011;364.

For peer review only

30. Hodgson C, Bellomo R, Berney S, et al. Early mobilization and recovery in mechanically ventilated patients in the ICU: a bi-national, multi-centre, prospective cohort study. *Crit Care* 2015;19:81.

31. Nydahl P, Ruhl AP, Bartoszek G, et al. Early mobilization of mechanically ventilated patients: a 1-day point-prevalence study in Germany. *Crit Care Med* 2014;42.

32. Jolley SE, Moss M, Needham DM, et al. Point Prevalence Study of Mobilization Practices for Acute Respiratory Failure Patients in the United States. *Crit Care Med* 2017;45(2):205-15.

33. Elliott D, McKinley S, Alison J, et al. Health-related quality of life and physical recovery after a critical illness: a multi-centre randomised controlled trial of a home-based physical rehabilitation program. *Crit Care* 2011;15(3):R142.

34. Berney S, Haines K, Skinner EH, et al. Safety and feasibility of an exercise prescription approach to rehabilitation across the continuum of care for survivors of critical illness. *Phys Ther* 2012;92(12):1524-35.

For peer review only

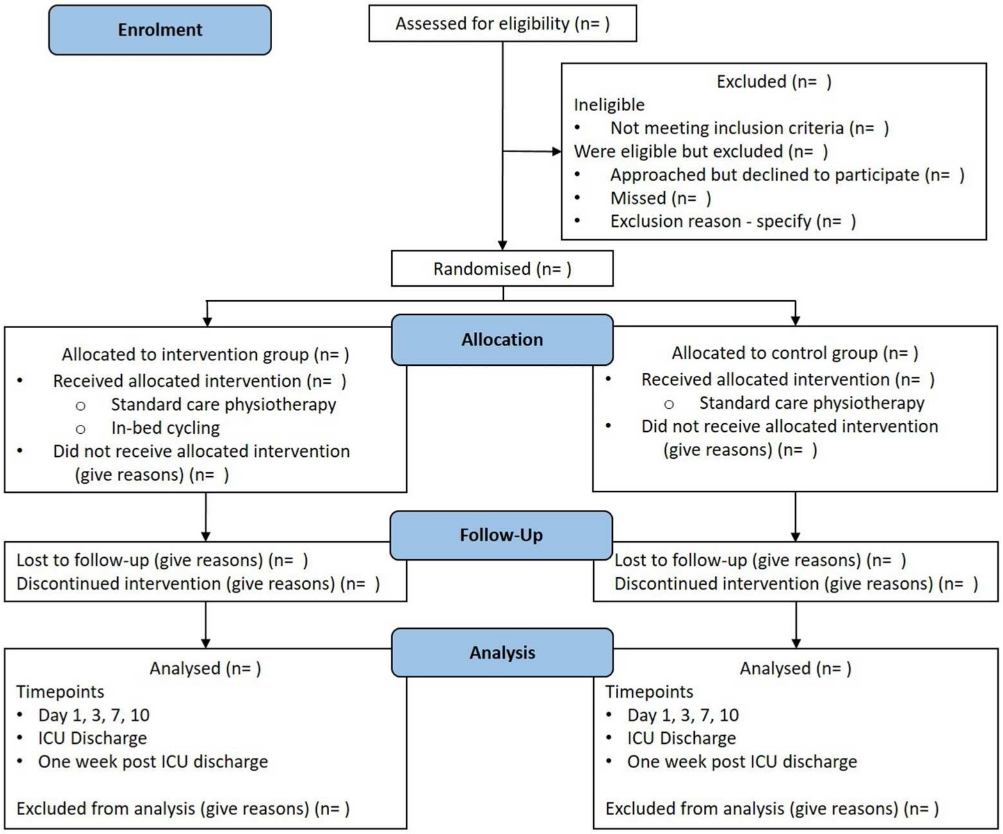

Figure 1

339x282mm (96 x 96 DPI)

SPIRIT
STANDARD PROTOCOL ITEMS: RECOMMENDATIONS FOR INTERVENTIONAL TRIALS

SPIRIT 2013 Checklist: Recommended items to address in a clinical trial protocol and related documents*

Section/item	Item No	Description	Location in manuscript
**Administrative information**			
Title	1	Descriptive title identifying the study design, population, interventions, and, if applicable, trial acronym	Title, page 1
Trial registration	2a	Trial identifier and registry name. If not yet registered, name of intended registry	Abstract, page 2
	2b	All items from the World Health Organization Trial Registration Data Set	Throughout manuscript
Protocol version	3	Date and version identifier	Additional information, page 15
Funding	4	Sources and types of financial, material, and other support	Additional information, page 14
Roles and responsibilities	5a	Names, affiliations, and roles of protocol contributors	Additional information, page 14
	5b	Name and contact information for the trial sponsor	Additional information page 14
	5c	Role of study sponsor and funders, if any, in study design; collection, management, analysis, and interpretation of data; writing of the report; and the decision to submit the report for publication, including whether they will have ultimate authority over any of these activities	Additional information, page 14

For peer review only

	5d	Composition, roles, and responsibilities of the coordinating centre, steering committee, endpoint adjudication committee, data management team, and other individuals or groups overseeing the trial, if applicable (see Item 21a for data monitoring committee)	Trial Management, page 12
**Introduction**			
Background and rationale	6a	Description of research question and justification for undertaking the trial, including summary of relevant studies (published and unpublished) examining benefits and harms for each intervention	Introduction, page 3
	6b	Explanation for choice of comparators	Introduction, page 3
Objectives	7	Specific objectives or hypotheses	Objectives, page 5
Trial design	8	Description of trial design including type of trial (eg, parallel group, crossover, factorial, single group), allocation ratio, and framework (eg, superiority, equivalence, noninferiority, exploratory)	Methods, Study Design and Setting, page 5
**Methods: Participants, interventions, and outcomes**			
Study setting	9	Description of study settings (eg, community clinic, academic hospital) and list of countries where data will be collected. Reference to where list of study sites can be obtained	Methods, Study Design and Setting, page 5
Eligibility criteria	10	Inclusion and exclusion criteria for participants. If applicable, eligibility criteria for study centres and individuals who will perform the interventions (eg, surgeons, psychotherapists)	Table 1, Inclusion and exclusion criteria, page 6
Interventions	11a	Interventions for each group with sufficient detail to allow replication, including how and when they will be administered	Study Intervention, page 6-7

For peer review only

	11b	Criteria for discontinuing or modifying allocated interventions for a given trial participant (eg, drug dose change in response to harms, participant request, or improving/worsening disease)	Table 2, Safety Guidelines, page 7
	11c	Strategies to improve adherence to intervention protocols, and any procedures for monitoring adherence (eg, drug tablet return, laboratory tests)	Study Intervention, page 7
	11d	Relevant concomitant care and interventions that are permitted or prohibited during the trial	Study Intervention, page 7
Outcomes	12	Primary, secondary, and other outcomes, including the specific measurement variable (eg, systolic blood pressure), analysis metric (eg, change from baseline, final value, time to event), method of aggregation (eg, median, proportion), and time point for each outcome. Explanation of the clinical relevance of chosen efficacy and harm outcomes is strongly recommended	Outcomes, page 8<br>Table 3
Participant timeline	13	Time schedule of enrolment, interventions (including any run-ins and washouts), assessments, and visits for participants. A schematic diagram is highly recommended (see Figure)	Figure 1
Sample size	14	Estimated number of participants needed to achieve study objectives and how it was determined, including clinical and statistical assumptions supporting any sample size calculations	Sample size, page 9
Recruitment	15	Strategies for achieving adequate participant enrolment to reach target sample size	Trial Management page 12.
**Methods: Assignment of interventions (for controlled trials)**			

Allocation:			
Sequence generation	16a	Method of generating the allocation sequence (eg, computer-generated random numbers), and list of any factors for stratification. To reduce predictability of a random sequence, details of any planned restriction (eg, blocking) should be provided in a separate document that is unavailable to those who enrol participants or assign interventions	Randomisation, page 6
Allocation concealment mechanism	16b	Mechanism of implementing the allocation sequence (eg, central telephone; sequentially numbered, opaque, sealed envelopes), describing any steps to conceal the sequence until interventions are assigned	Randomisation, page 6
Implementation	16c	Who will generate the allocation sequence, who will enrol participants, and who will assign participants to interventions	Randomisation, page 6
Blinding (masking)	17a	Who will be blinded after assignment to interventions (eg, trial participants, care providers, outcome assessors, data analysts), and how	Data collection, page 10-11
	17b	If blinded, circumstances under which unblinding is permissible, and procedure for revealing a participant's allocated intervention during the trial	Data collection, page 10-11
**Methods: Data collection, management, and analysis**			

Data collection methods	18a	Plans for assessment and collection of outcome, baseline, and other trial data, including any related processes to promote data quality (eg, duplicate measurements, training of assessors) and a description of study instruments (eg, questionnaires, laboratory tests) along with their reliability and validity, if known. Reference to where data collection forms can be found, if not in the protocol	Data collection, page 9-11
	18b	Plans to promote participant retention and complete follow-up, including list of any outcome data to be collected for participants who discontinue or deviate from intervention protocols	Study Intervention, page 7
Data management	19	Plans for data entry, coding, security, and storage, including any related processes to promote data quality (eg, double data entry; range checks for data values). Reference to where details of data management procedures can be found, if not in the protocol	Data collection, page 9-11
Statistical methods	20a	Statistical methods for analysing primary and secondary outcomes. Reference to where other details of the statistical analysis plan can be found, if not in the protocol	Analysis, page 11
	20b	Methods for any additional analyses (eg, subgroup and adjusted analyses)	Analysis, Table 5, page 11-12
	20c	Definition of analysis population relating to protocol non-adherence (eg, as randomised analysis), and any statistical methods to handle missing data (eg, multiple imputation)	Analysis, page 11
**Methods: Monitoring**			

Data monitoring	21a	Composition of data monitoring committee (DMC); summary of its role and reporting structure; statement of whether it is independent from the sponsor and competing interests; and reference to where further details about its charter can be found, if not in the protocol. Alternatively, an explanation of why a DMC is not needed	Contributors, page 14
	21b	Description of any interim analyses and stopping guidelines, including who will have access to these interim results and make the final decision to terminate the trial	Trial management, page 12
Harms	22	Plans for collecting, assessing, reporting, and managing solicited and spontaneously reported adverse events and other unintended effects of trial interventions or trial conduct	Trial management, page 12
Auditing	23	Frequency and procedures for auditing trial conduct, if any, and whether the process will be independent from investigators and the sponsor	Trial management, page 12
**Ethics and dissemination**			
Research ethics approval	24	Plans for seeking research ethics committee/institutional review board (REC/IRB) approval	Abstract, page 2
Protocol amendments	25	Plans for communicating important protocol modifications (eg, changes to eligibility criteria, outcomes, analyses) to relevant parties (eg, investigators, REC/IRBs, trial participants, trial registries, journals, regulators)	Analysis, page 11
Consent or assent	26a	Who will obtain informed consent or assent from potential trial participants or authorised surrogates, and how (see Item 32)	Consent, page 5-6

w only

	26b	Additional consent provisions for collection and use of participant data and biological specimens in ancillary studies, if applicable	Not applicable
Confidentiality	27	How personal information about potential and enrolled participants will be collected, shared, and maintained in order to protect confidentiality before, during, and after the trial	Data Collection, page 10
Declaration of interests	28	Financial and other competing interests for principal investigators for the overall trial and each study site	Additional information, page 14
Access to data	29	Statement of who will have access to the final trial dataset, and disclosure of contractual agreements that limit such access for investigators	Additional information, page 14-15
Ancillary and post-trial care	30	Provisions, if any, for ancillary and post-trial care, and for compensation to those who suffer harm from trial participation	Additional information, page 15
Dissemination policy	31a	Plans for investigators and sponsor to communicate trial results to participants, healthcare professionals, the public, and other relevant groups (eg, via publication, reporting in results databases, or other data sharing arrangements), including any publication restrictions	Dissemination, page 12
	31b	Authorship eligibility guidelines and any intended use of professional writers	Dissemination, page 12
	31c	Plans, if any, for granting public access to the full protocol, participant-level dataset, and statistical code	Dissemination, page 12
**Appendices**			

For peer review only

Informed consent materials	32	Model consent form and other related documentation given to participants and authorised surrogates	Substitute Decision Maker Consent Form
Biological specimens	33	Plans for collection, laboratory evaluation, and storage of biological specimens for genetic or molecular analysis in the current trial and for future use in ancillary studies, if applicable	Not applicable

*It is strongly recommended that this checklist be read in conjunction with the SPIRIT 2013 Explanation & Elaboration for important clarification on the items. Amendments to the protocol should be tracked and dated. The SPIRIT checklist is copyrighted by the SPIRIT Group under the Creative Commons "Attribution-NonCommercial-NoDerivs 3.0 Unported" license.

For peer review only

BMJ Open

Critical Care Cycling Study (CYCLIST) Trial Protocol: a randomised controlled trial of usual care versus usual care plus additional in-bed cycling sessions in the critically ill

Journal:	*BMJ Open*
Manuscript ID	bmjopen-2017-017393.R1
Article Type:	Protocol
Date Submitted by the Author:	05-Jul-2017

Complete List of Authors:	Nickels, Marc; Princess Alexandra Hospital, Physiotherapy Department<br>Aitken, Leanne M.; City, University of London, School of Health Sciences; Griffith University Menzies Health Institute Queensland, National Centre of Research Excellence in Nursing Interventions for Hospitalised Patients<br>Walsham, James; Princess Alexandra Hospital, Intensive Care Unit; University of Queensland<br>Barnett, Adrian; Queensland University of Technology, Institute of Health and Biomedical Innovation<br>McPhail, Steven; Queensland University of Technology, Institute of Health and Biomedical Innovation; Metro South Hospital and Health Service, Centre for Functioning and Health Research

**Primary Subject Heading**:	Intensive care
Secondary Subject Heading:	Rehabilitation medicine
Keywords:	INTENSIVE & CRITICAL CARE, REHABILITATION MEDICINE, cycle ergometry, muscle atrophy, physiotherapy, critical illness

SCHOLARONE™
Manuscripts

For peer review only

Critical Care Cycling Study (CYCLIST) Trial Protocol: a randomised controlled trial of usual care versus usual care plus additional in-bed cycling sessions in the critically ill

Authors

1. Marc R Nickels [a, *]
2. Leanne M Aitken [b, c, d]
3. James Walsham [d, e]
4. Adrian G Barnett [f]
5. Steven M McPhail [f, g]

a) Intensive Care Unit and Physiotherapy Department, Princess Alexandra Hospital, Brisbane, Australia

b) School of Health Sciences, City, University of London, London, United Kingdom

c) National Centre of Research Excellence in Nursing Interventions for Hospitalised Patients, Menzies Health Institute Queensland, Griffith University, Brisbane, Australia

d) Intensive Care Unit, Princess Alexandra Hospital, Brisbane, Australia

e) University of Queensland, Brisbane, Australia

f) School of Public Health & Social Work and Institute of Health and Biomedical Innovation, Queensland University of Technology, Brisbane, Australia

g) Centre for Functioning and Health Research, Metro South, Brisbane, Australia

* Corresponding author at: Intensive Care Unit and Physiotherapy Department, Princess Alexandra Hospital, Brisbane, Australia, 4102. Tel.: +61 7 3176 2401. E-mail address: marc.nickels@health.qld.gov.au (M. Nickels).

Keywords:

Critical illness; Cycle ergometry; Intensive care units; Muscle weakness; Rehabilitation

For peer review only

ABSTRACT

Introduction

In-bed cycling with critically ill patients has been shown to be safe, feasible and improve physical function outcomes at hospital discharge. The effect of early in-bed cycling on reducing the rate of skeletal muscle atrophy, and associations with physical and cognitive function are unknown.

Methods and analysis

A single centre randomised controlled trial in a mixed medical-surgical intensive care unit (ICU) will be conducted. Adult patients (n= 68) who are expected to be mechanically ventilated for more than 48 hours and remain in ICU for a further 48 hours from recruitment will be randomly allocated into either (1) a usual care group or (2) a group that receives usual care and additional in-bed cycling sessions. The primary outcome is change in rectus femoris cross-sectional area at day 10 in comparison to baseline measured by blinded assessors. Secondary outcome measures include muscle strength, incidence of ICU acquired weakness, handgrip strength, time to achieve functional milestones (sitting out of bed, walking), Functional Status Score in ICU, ICU mobility scale, six-minute walk test one week post ICU discharge, incidence of delirium and quality of life (EQ-5D-5L). Quality of life assessments will be conducted at day 10 post ICU admission and 3 and 6 months post hospital discharge. Participants in the intervention group will complete an acceptability of intervention questionnaire.

Ethics and dissememination

Appropriate ethical approval from Metro South Health Human Research Ethics Committee has been attained. Results will be published in peer-reviewed publications and presented at scientific conferences to assist planning of future multi-centre randomised controlled trials (if indicated) that will test in-bed cycling as an intervention to improve the physical, cognitive and health-related quality of life outcomes of critically ill patients.

Trial Registration

This trial has been prospectively registered on the Australian and New Zealand Clinical Trial Registry (ACTRN12616000948493).

For peer review only

Strengths and Limitiations of this study

- The randomised trial design with blinded assessments of skeletal muscle size, strength and function to provide objective measures of difference
- The inclusion of an acceptability questionnaire will provide useful insights for subsequent implementation (if indicated)
- The study may not be powered for all secondary outcomes, and evidence of effect size from pilot data is not available for those measures

For peer review only

Background and Rationale

Critically ill patients often require mechanical ventilation for periods greater than 48 hours. It has been identified that skeletal muscle wasting occurs early and rapidly during the first week of critical illness [1]. Despite international recommendations for critically ill patients to commence activity as early as possible [2] it has been identified that exercise interventions are rarely initiated when a patient is on mechanical ventilation [3]. This leads to prolonged immobility and may contribute to the development of intensive care unit acquired weakness (ICUAW) [4]. In-bed cycling using a cycle ergometer has been proposed as a safe and feasible method of introducing early exercise for critically ill patients on mechanical ventilation who are sedated and immobile, this includes patients requiring inotropic support [5-7]. In-bed cycling may also assist in the preservation of muscle architecture. To date only one randomised controlled trial (RCT) utilising in-bed cycling in the critically ill population has been published [8]. This single centre RCT (n = 90) conducted in Belgium, found in-bed cycling to be safe and to improve critically ill patients' 6-minute walk distance, quadriceps force and Short Form-36 (SF-36) physical function scores on hospital discharge [8]. A limitation of this study was that the effects of the intervention at the muscular level were not assessed with muscle biopsy or ultrasound.[8] A pilot case-matched study of in-bed Functional Electrical Stimulated (FES) cycling intervention in addition to usual care, found positive physical outcomes observed among the cycling group including less time required to achieve functional milestones, time to stand and time to ambulate independently [9]. In addition, a shorter duration of delirium among those who participated in the Functional Electrical Stimulation in-bed cycling intervention was observed [9].

A recent clinical trial has demonstrated that critically ill patients may experience persistent weakness despite participating in intensive exercise programs whilst they are critically ill [10]. It has been suggested that intensive exercise programs may not be effective if the commencement of these programs is delayed [11]. Early exercise commencement is intended to assist in the maintenance of muscle mass. This may be achieved through a moderation of the inflammatory process [11]. Consequently, the effectiveness of exercise interventions that can commence early during critical illness are necessary to demonstrate if patient outcomes are improved by the early commencement of exercise during a period of critical illness.

Early clinical studies in the field have demonstrated potential for in-bed cycling interventions (with and without Functional Electrical Simulation) to improve physical and cognitive function among critically ill patients [8 9]. There are currently no published RCTs investigating the effectiveness of in-bed cycling in critically ill patients requiring prolonged mechanical ventilation on quadriceps structure, ICU-acquired weakness and cognitive outcomes. Consequently, further investigation of

For peer review only

the effect of in-bed cycling on muscle structure, physical function and cognitive function is warranted.

Objectives

The objectives of this study are to:

(1) Examine whether in-bed cycling in addition to usual care is effective in reducing the rate of rectus femoris cross-sectional area (CSA) atrophy and ICUAW in patients requiring more than 48 hours of invasive mechanical ventilation compared with usual care.

(2) Investigate if in-bed cycling in addition to usual care is associated with better functional and cognitive outcomes in patients predicted to require more than 48 hours of invasive mechanical ventilation compared to usual care.

METHODS

Study Design and Setting

This trial will be a two arm, parallel randomised controlled trial with individual participant allocation and blinding of the primary outcome assessor. It will be conducted in a 25-bed tertiary mixed medical and surgical adult intensive care unit in Brisbane, Australia. Participants will be allocated 1:1 to receive either usual care or in-bed cycling in addition to usual care (Figure 1). In designing this study, the SPIRIT 2013 Checklist was utilised to ensure that all recommended items in a clinical trial were addressed [12].

Consent

It is anticipated that most patients who are eligible to participate in this study in the ICU setting will not be able to provide informed consent at the time of study enrolment. For those patients that may have the capacity to provide informed consent, the Richmond Agitation and Sedation Scale (RASS) will be used to determine if a patient is rated 'Alert and Calm'. If a patient is rated as 'Alert and Calm' on the RASS, the Confusion Assessment Method for the ICU (CAM-ICU) will be used to determine if a patient has had delirium within the preceding 24 hours. Provided a patient passes the RASS and CAM-ICU assessments the treating clinical team will be approached to determine if the patient has the capacity to provide informed consent. Patients without delirium for the preceding 24 hours and deemed to have capacity will be approached to provide their own written informed consent for study participation. For eligible patients considered unable to provide informed consent at the time of study enrolment, substitute decision makers (family members or next of kin), will be approached for written informed consent. The Queensland Civil and Administrative Tribunal (QCAT) have approved an application to provide consent for individuals who are unable to give consent and do

not have a next of kin that is accessible to request consent on the patients' behalf. Delayed consent from the patient will be sought once they can provide consent for themselves if they are enrolled using QCAT approval. Participation in the study is voluntary. Patients or their substitute decision makers are able to withdraw at any time without any negative consequences on the care they would receive, their ongoing relationship with the hospital, or staff involved in their care.

The substitute decision maker consent form (PICF_SDM_CYCLIST_V3.0_20160701) is attached as a supplementary file.

Randomisation

Patients will be individually randomised in a 1:1 ratio to either intervention or usual care group. Blocking (random block sizes) will be used to help balance the groups. An investigator not involved in the screening, consenting, allocation or assessment processes will use computerised random number generation to create the randomisation sequence. A randomisation sequence will be uploaded onto the Research Electronic Data Capture (REDCap) secure web based computer application [13]. The REDCap randomisation module will reveal the group allocation of each patient to the intervention coordinator after a patients' baseline data has been collected.

Participants

The study aims to recruit 68 participants. Adult patients expected to require at least 48 hours of mechanical ventilation will be recruited, with the inclusion and exclusion criteria listed in Table 1. Criteria to guide when to discontinue or not deliver an intervention are listed in Table 2. Participants can be recruited into the study and baseline sonography measures performed if a patient does not curently meet the in-bed cycling safety criteria in Table 2 (but are considered by their treating clinical team to be likely to meet the criteria within their stay in ICU).

Table 1 Inclusion and exclusion criteria

Inclusion	Exclusion
Expected to require more than 48 hours of invasive mechanical ventilation	Pre-existing condition that is likely to impair mobility/ mobility assessment (i.e. significant neurological, musculoskeletal, cognitive or mental health disorder)
Able to provide consent or have a family member consent on their behalf	Neuromuscular disorder or acute primary brain lesion (i.e. traumatic brain injury, intracranial haemorrhage, stroke, or hypoxic brain injury)
Enrolled into the study within 96 hours of ICU admission	Injuries precluding cycle ergometry (i.e. spinal / pelvic / lower limb orthopaedic injuries / open abdominal wound)
Expected to remain in ICU for more than 48 hours following study enrolment	Obesity > 135 kilograms (MOTOmed Letto 2 maximum rated weight capacity)
	Uncontrolled seizures or status epilepticus
	Dire prognosis (i.e. unlikely to survive the current admission)

For peer review only

	Pregnancy
	Children and/or young people (i.e. < 18 years)

Study Intervention

All study patients will receive usual physiotherapy interventions whilst in intensive care. Physiotherapy interventions will include (but are not limited to): respiratory physiotherapy, physical rehabilitation exercise interventions including sitting on the edge of the bed, sit to stand transfers, sitting out of bed and walking. Usual care physiotherapy interventions will be prioritised over the in-bed cycling intervention. Safety guidelines (see Table 2) will be used to determine if the intervention group patients are able to complete an additional daily 30-minute progressive lower limb in-bed cycling using a bedside cycle ergometer (MOTOmed Letto 2). The lead physiotherapist (MRN) who has over 10 years of experience in rehabilitative exercise with critically ill patients will primarily conduct the majority of in-bed cycling sessions. He has been trained by industry cycle ergometry representatives and has over 5 years of experience conducting in-bed cycling sessions with critically ill patients. Experienced ICU physiotherapists trained in conducting in-bed cycling sessions may conduct the in-bed cycling sessions if the lead physiotherapist is unavailable. During the in-bed cycling exercise interventions the patients' vital signs will be monitored. If the intervention group are in a state of low arousal or sedated they will cycle continuously and passively at the default passive speed of the cycle ergometer (20 revolutions per minute) for 30 minutes. When the participant is following commands, the clinician will verbally encourage the patient to complete in-bed cycling sessions actively for a duration of 30 minutes. Once the patient can cycle actively the resistance applied by the cycle ergometer will be adjusted to facilitate patient intensity of between 3 and 5 using the visual Borg scale rate of perceived exertion (category ratio 10),[14] within the specified safety guidelines. An exercise intensity of 3-5 on the Borg rate of perceived exertion scale has been shown to be safe and feasible with critically ill patients.[15] This will enable the patient to cycle in-bed either passively or actively with assistance from the cycle ergometer. If a patient unexpectedly commences active cycling the additional active in-bed cycling safety criteria will become relevant. If the patient is deemed unsuitable to continue active in-bed cycling they will be asked to resume passive cycling. If the patient continues to actively cycle the session will be ceased. The in-bed cycling sessions will continue until the patient completes a minimum of 5 in-bed cycling sessions, unless the patient is discharged from hospital prior to completing 5 sessions. The intervention will continue in the acute hospital ward if the patient is discharged from ICU prior to completing 5 in-bed cycling sessions. Whilst the patient remains in ICU, in-bed cycling sessions will continue (up to 7 days per week), up to 28 days' post ICU admission. This frequency is congruent with usual physiotherapy services that can provide rehabilitative exercise interventions to ICU patients up to seven days per week. Patients

For peer review only

randomised to the usual care arm do not routinely complete in-bed cycling sessions during their hospitalisation. Any deviations from the planned protocol will be recorded to enable appropriate intervention description and if indicated a per-protocol analysis (in addition to the primary intention to treat analysis).

Participants will not be coerced to complete any intervention or outcome measure. Participants' may be discharged home from the participating acute hospital before they have completed outcome measures at each assessment time-point. If this occurs, participants will be asked to return to the hospital to enable the remaining outcome measures to be completed and expenses related to taxi or parking costs will be reimbursed.

Table 2 Safety guidelines

**Active or passive exercise should not be delivered if:**
Clinician opinion that patient condition unstable
Resting HR < 40 or > 120 bpm or new arrhythmia
Evidence of coronary ischaemia e.g. chest pain or ECG changes
MAP < 60 or SBP > 200 mmHg
SpO2 < 90%
RASS ≥ 2
Wounds of leg, pelvis or lumbar spine precluding cycle ergometry
Evidence of active bleeding or coagulation disorder: INR >1.8, PLT <50,000/microL. *
Femoral vascular access ** e.g. dialysis catheter, IABP, ECMO or lower limb arterial line ***
Acute DVT or PE
**Active exercise should not be delivered if:**
> 20 µg/min of noradrenaline or comparable inotropic or vasopressor support
FiO2 > 0.55 or PEEP > 10 cmH2O
RR > 30 with adequate ventilatory support
Temperature > 39° Celsius
**Stopping criteria: active or passive exercise should cease if:**
HR < 50 or > 140 bpm, or new arrhythmia develops (including ventricular ectopic or new-onset AF)
Evidence of coronary ischaemia e.g. chest pain or ECG changes
MAP < 60 mmHg
SBP > 200 mmHg
Clinical signs of cardiorespiratory distress
SpO2 < 90% for more than 1 minute
Patient request to stop therapy

HR, heart rate; bpm, beats per minute; ECG, electrocardiogram; FIO2, fraction of inspired oxygen; MAP, mean arterial pressure; SBP, systolic blood pressure; DBP, diastolic blood pressure; SpO2, saturation of peripheral oxygen; RASS, Richmond Agitation Sedation Score; INR, international normalised ratio; PLT, platelets; microL, microlitre; IABP, intra-aortic balloon pump; ECMO, extracorporeal membrane oxygenation; DVT, deep vein thrombosis; PE, pulmonary embolism; PEEP, positive end expiratory pressure; RR, respiratory rate; AF, atrial fibrillation

** Values outside this range would be tolerated if patient therapeutically anticoagulated*

*** Other than femoral central line*

****If a femoral vascular access is inserted unilaterally the contralateral leg may be cycled unilaterally.*

Outcomes

Table 3 provides a summary of the outcome measures. The primary outcome is the percentage of change in rectus femoris CSA measured at baseline (within 24 hours of study enrolment) and day 10 (post study enrolment), measured by a blinded assessor. The participants will be assessed to examine whether there is a between group difference in the amount of rectus femoris CSA atrophy. The authors anticipate that less rectus femoris CSA atrophy will be observed among the in-bed cycling group. Day 10 post study enrolment has been chosen as the primary endpoint to enable comparison with previously published data on acute skeletal muscle wasting in patients with critical illness and consistent with this previously reported time-frame of observed muscle wasting.[1] Secondary outcome measures in the CYCLIST study are muscle strength, physical function, cognition, quality of life, and acceptability of intervention.

Table 3 Descriptions of outcome measures for CYCLIST RCT

Assessment Component	Outcome Measure	Description
Muscle Morphology	Ultrasound	RF CSA, AP thickness of RF and VI. Measured in triplicate on right anterior thigh one third distance from superior patella to ASIS Patient positioned in supine, thirty degress head elevation [16]
Muscle Strength	MRC Sum Score	Standardised sum of twelve MMTs, three MMTs per limb Score ≤ 48 indicative of ICU acquired weakness [17]
	Handgrip strength dynamometry	Triplicate bilateral measurement using a Jamar Digital Dynamometer (Lafayette) with seated patient [18]
Physical Function	ICU Mobility Scale	Best level of function achieved in ICU using an eleven-point ordinal scale [19]
	FSS-ICU	Patients' function measured an eight-point ordinal scale [20 21]
	Functional milestones	Time to achieve functional milestones: Sit out of bed, time to stand, mobilise with assistance and mobilise independently
	6-minute walk test	Sub-maximal endurance test of distance walked by a patient in six minutes [22]
Cognition	CAM-ICU	Incidence and recorded episodes of acute delirium [23]
Quality of Life	EQ-5D-5L	EuroQol five dimensions questionnaire five level scale [24]
Intervention Acceptability	Customised questionnaires	Questionnaire about the acceptability of the in-bed cycling intervention

RF, rectus femoris; CSA, cross sectional area; VI, vastus intermedius; AP, anterior posterior; ASIS, anterior superior illiac crest; MRC, Medical Research Council; MMT, manual muscle test; ICU, intensive care unit; FSS-ICU, Funcitonal Status Score for the Intensive Care Unit; CAM-ICU Confusion Assessment Measure for the Intensive Care Unit; EQ-5D-5L, EuroQol five dimensions questionnaire five level scale.

Demographic information such as age, gender and diagnostic code will be collected. Illness related information including length of mechanical ventilation, ICU (LOS) and hospital LOS and discharge destination, illness severity (APACHE III [25], Sequential Organ Failure Assessment (SOFA) [26] score), pre-

For peer review only

morbid co-morbidities, neuromuscular blockade and sedation medications administered, nutrition received, cumulative fluid balance, patient height and weight, and body mass index will also be collected.

Sample Size

Based on the repeated measures design this study will have 80% power to detect a between groups difference of 2.9% on our primary outcome (change in rectus femoris CSA) between the intervention and usual care participants, (assuming type I error 0.05, standard deviation of 6%, and within-patient correlation of 0.5 between assessments, and after accounting for an up to 20% drop out rate including in-hospital mortality). A total of 68 patients (34 in each group) will be recruited into the study. With a drop-out rate of up to 20%, this would represent approximately 27 participants completing the trial in each group. It is noteworthy that some of the assumptions for this calculation have been made in the absence of prior randomised control trial data. There is some risk that the observed trial data may not fit these assumptions (e.g., discrepancies in standard deviations or within-patient correlation between the repeated measures), and the power of the study may differ from this a-priori sample size estimate.

Data Collection

The study data will be collected at the time points summarised in Table 4.

Table 4 CYCLIST summary of timepoints of assessments

	Baseline	Day 3	Day 7	Day 10	ICU discharge	1 week post ICU discharge*	3, 6 months' post hospital discharge
Severity of illness							
APACHE III	✓						
SOFA	✓	✓	✓	✓	✓		
Muscle Morphology							
Quadriceps Ultrasonography	✓	✓	✓	✓		✓	
Strength Measures							
MRC Sum Score					✓	✓	
Handgrip dynamometry					✓	✓	
Physical Function Measures							
ICU Mobility Scale					✓		
FSS-ICU					✓		
Functional Milestones	✓	✓	✓	✓	✓	✓	
6-MWT						✓	
Cognition							
CAM-ICU	✓	✓	✓	✓	✓		
Quality of Life							
EQ-5D-5L				✓			✓

For peer review only - http://bmjopen.bmj.com/site/about/guidelines.xhtml

**Acceptability of Intervention**			
	✓**		
**Patient Outcomes**			
ICU Length of Stay	✓		
Hospital Length of Stay		✓***	
Acute discharge destination		✓***	
Mortality	✓	✓***	✓

ICU, intensive care unit; APACHE, Acute Physiology and Chronic Health Evaluation; SOFA, Sequential Organ Failure Assessment score; MRC, Medical Research Council; FSS-ICU, functional status score in the intensive care unit; CAM-ICU, confusion assessment measure in intensive care; EQ-5D-5L, EuroQol five dimensions. questionnaire five level scale.

** 1 week post ICU discharge or at acute hospital discharge if sooner.*

*** At completion of in-bed cycle ergometry sessions*

**** Measured at acute hospital discharge*

The management of study data will be dependent on the type of data collected. Baseline data will be entered directly into Research Electronic Data Capture (REDCap) designed digital clinical trial workflow management software [13]. Ultrasound results will be uploaded onto the secure hospital based AGFA IMPAX 6.5.3.1005 Medical Image Viewer application. In-bed cycling session data, physical assessment measures, acceptability of in-bed cycling intervention and quality of life questionnaires will be recorded initially onto research data sheets. All information recorded on data sheets will be subsequently entered into the REDCap application.

Assessments of muscle size will be assessed by ultrasound and performed by registered post-graduate trained sonographers with expertise in musculoskeletal sonography. Sonographers' will be blinded to patient treatment group allocation and will be responsible for analysing and scoring all ultrasound images. The study sonographers were involved in the development and standardisation of the ultrasound procedure prior to the commencement of the study.

Assessment of muscle strength and function will be completed by cardiorespiratory physiotherapy assessors with a minimum of 3 years' experience at participants' ICU discharge and 7-days post ICU discharge. These physiotherapists will be blinded to group allocation. The same physiotherapists will also assess 6-minute walk test distance 7-days following ICU discharge. The study physiotherapists were trained in the standardised assessment of the study outcome measures prior to the commencement of the study.

A physiotherapist not involved in blinded outcome assessment will be responsible for conducting the in-bed cycling sessions, and the remaining 'usual care' physiotherapy will be completed by hospital

For peer review only

department physiotherapists not involved in the study. Additionally, the primary statistician will be blinded to treatment group allocation.

To minimise the chances of unintentional unblinding, at the beginning of each assessment the blinded assessors will clearly state that they wish to complete the assessment process without knowing which group the patient was allocated too. If any blinded assessors were to become unblinded to group allocation for any patient (e.g., through inadvertent revelation by a patient being assessed or unanticipated exposure to a patient taking part in an in-bed cycling session), they have been instructed to report this to the intervention coordinator (MRN) who will also record when this occurred.

ANALYSIS

Data will be analysed and reported using intention to treat principles (primary analysis). A per protocol analysis will be conducted to assist in determining the efficacy of the in-bed cycling protocol if variation from the planned protocol occurs for a substantial proportion of patients. The per protocol analysis will include only patients who adhered to the protocol and received at least 80% of training sessions (minimum of 4 sessions).

Descriptive statistics and generalized linear mixed models will be used to examine the effect of group allocation (intervention vs. usual care) on the primary and secondary outcomes. As this is a randomised trial we do not plan to adjust for potential confounders (e.g., age, gender, comorbidities), but will compare the characteristics of the sample by treatment group and may adjust if a potential confounder differs greatly between groups.

If blinded sonographers are unable to complete an ultrasound measurement at designated time-points, then the scan will be performed within a day (± 1 day) of the designated time-point. A subsequent sensitivity analysis will be conducted to determine if there is a difference by measurement timing. If the primary outcome cannot be collected by a sonographer within the specified timeframe a physiotherapist experienced in musculoskeletal ultrasound will measure thigh muscle size. The measurement will be from the most anterior aspect of rectus femoris to the anterior surface of the femur. This will assist in the imputation of the missing primary outcome data. Statistical analysis of available data will be used for the primary analysis when data is completely missing. Multiple imputation will be conducted if more than 20% of outcome data is missing. Additional planned secondary analyses are listed in Table 5. The principal investigators will

For peer review only - http://bmjopen.bmj.com/site/about/guidelines.xhtml

determine if study protocol modifications are required and any modifications to the existing protocol will be declared.

Table 5 Planned secondary analyses

Muscle wasting at baseline, day 3, day 7 and 1 week post ICU discharge
Muscle wasting adjusted for number of failing vital organs *
Muscle wasting adjusted for severity of illness on admission to ICU **
Muscle wasting adjusted for the number of days prior to a patient commencing active activity
Muscle wasting adjusted for the patients' cumulative fluid balance on the day of the ultrasound scan
Relationship between muscle wasting and participants' nutritional intake whilst in ICU
Relationship between sedative and paralytic medications and muscle wasting
Cost comparision of hospitalisation for both the intervention and usual care groups ***
Hospital readmissions post acute hospital discharge over a two year time period ****
Mortality *****

Utilising: * Sequential Organ Failure Score (SOFA), ** APACHE III data, *** health service utilisation data, **** Queensland Hospital Admitted Patient Data Collection (QHAPDC), ***** Queensland Health Statistical Services Branch

TRIAL MANAGEMENT

The principal investigator (MRN) will oversee the conduct and progress of the trial. The principal investigator will screen the daily admission to ICU lists and liaise with the treating medical teams to optimise participant enrolment. No interim analyses are planned. The principal investigator will ensure all research personnel are appropriately orientated and trained, oversee recruitment and report to a trial safety monitoring committee who will monitor the progress and conduct of the trial. The trial safety monitoring committee will include: a physiotherapist and researcher experienced in the safe conduct of clinical trials with physiotherapy interventions; the trial coordinator and principal investigator; a critical care nurse who is also experienced in the safe conduct of clinical trials in critical care settings and two ICU medical consultants experienced with the safe conduct of clinical trials in intensive care units. One of the ICU medical consultants is employed externally to the study site. The principal investigator will provide an update report to the Safety Monitoring Committee on a monthly basis (and additional ad-hoc reports if an adverse event occurs). Additionally any Serious Adverse Events will be reported to the approving Metro South Human Research Ethics Committee that is overseeing the study. During in-bed cycling sessions, participants will be monitored for adverse events that will be recorded on the session data collection form. Adverse events that are being monitored for are; line or airway dislodgement, increase in ventatory support that persist greater than five minutes post exericse (e.g. increase PEEP or FiO2), blood oxygen desaturation less than 88% for more than one minute, increase in vasoactive or pain relief medication greater than 5mcg/min, increase in systolic blood pressure greater than 180mmHg for more than two minutes, increase in heart rate greater than 140bpm for more than two minutes, decrease in mean arterial

For peer review only

blood pressure less than 60mmHg for greater than two minutes and decrease in heart rate less than 50bpm for more than two minutes.

DISSEMINATION

Study results will be disseminated via publication in peer-reviewed literature and scientific conference presentations. It is anticipated that media releases in lay form will be completed to target the general community. Study results will also be placed on a university website for viewing by participants and other interested parties. There are no publication restrictions. Authorship eligibility guidelines as outlined in The Australian Code for the Responsible Conduct of Research [27] and consistent with those proposed by the International Committee of Medical Journal Editors will be followed to determine authorship [28].

DISCUSSION

Survival rates following critical illness are improving [29]; however, patients are experiencing deficits in physical and cognitive function that do not equal age matched peers 5 years after an episode of critical illness [30]. The delayed initiation of rehabilitaitve exercise interventions with critically ill patients may explain the limited effectiveness of clinical trials that have studied the effect of exercise interventions on patients functional outcomes [11]. A binational clinical trial that aimed to commence exercise interventions as early as possible with mechanically ventilated patients reported that despite the presence of a dedicated early mobility team, patient mobilisation out of bed while mechanical ventilation was in-situ was rare [31]. These results are substantiated by point prevalance studies from Australia, New Zealand and Germany that report that in 1281 patient days only one patient with an endotracheal tube was mobilised out-of-bed [3 32]. The presence of an endotracheal tube is negatively correlated with out-of-bed mobilisation in the United States with a reported odds ratio of 0.1, [95% CI, 0.05-0.2] [33]. Studies have reported that following a period of critical illness, rehabilitative interventions do not hasten recovery when they are provided post acute hospital discharge [15 34]. Consequently this trial will provide valuable clinical trial evidence regarding the effect of an exercise intervention initiated early in the critically ill patient's illness. Specifically, it will report empirical data about the effect of the intervention on the rate of skeletal muscle wasting, and whether early exercise interventions that can be feasibly implemented among people who are mechanically ventilated are associated with improved physical, cogntive and health related quality of life outcomes.

CYCLIST is a Phase IIb randomised controlled trial that is powered to investigate if the early application of an additional in-bed cycling intervention is able to reduce the rate of skeletal muscle

For peer review only

atrophy of patients' quadriceps muscle during and immediately following a period of critical illness, in comparison to usual care. Secondary outcomes evaluated in this study will assist sample size calculations for future studies to assess for efficacy of functional outcomes. The study will also aid planning of future rehabilitation based clinical trials with regard to rate of participant recruitment. The investigators are planning to meet at the completion of the study to facilitate reflection on aspects of the study that could be improved and to consider whether a definitive Phase III RCT is warranted. This will include consideration of the evidence of effect on primary and secondary outcomes, participant recruitment and adherence to the in-bed cycling protocol and rate, as well as acceptability of the intervention.

The strengths of this study are the implementation of the in-bed cycling intervention as soon as feasible (including while patients are still mechanically ventilated), with clear commencement and stopping rules. Another strength of the study is the blinded assessment of muscle structure, strength and function assessments. Measurement of the effect of early in-bed cycling on quality of life post hospital discharge is another strength that will assist to demonstrate if potential gains made early during a period of critical illness correspond to lasting functional improvements. It is expected that this study will also provide insights regarding the feasibilty of the in-bed cycling intervention with critically ill patients from the rates of compliance and completion of the in-bed cycling exercise intervention. The acceptability of in-bed cycling intervention from the perspective of critically ill patients' will be sought through a questionnaire and provide new information to inform potential implementation strategies for this intervention. A potential limitation of the study may be difficulty completing functional assessment measures at ICU discharge and at one week post-ICU discharge with patients who are either profoundly weak or present with an acute cognitive dysfunction. Further, usual care physiotherapy interventions will be delivered by treating teams independent of the study and prioritised over in-bed cycling. The frequency, intensity, type and duration of all usual care physiotherapy interventions are not being prospectively recorded as part of the trial outcomes. As the study design is a randomised controlled trial it is anticipated that the usual care physiotherapy will be similar across groups. Also this study is being conducted at a single centre and therefore results may need to be interpreted will caution.

Word Count: 3875

For peer review only

Contributors

MRN contributed to study conception, design, grant acquisition, trial management (including intervention coordination, data collection), data management, protocol drafting, appraisal and editing. SMM, LMA and JW contributed to study conception, design, grant acquisition, analysis plan, data management, protocol drafting, appraisal and editing. AGB contributed to study analysis plan, grant acquisition, data management, protocol drafting, appraisal and editing. All authors read and approved the final manuscript.

Funding

This is an investigator initiated trial without external sponsors. Following a competitive peer review process Metro South Health Study, Education and Research Trust Account (SERTA) awarded this study a grant in 2015. MRN has also been awarded a competitive Princess Alexandra Research Support Scheme Postgraduate Scholarship to conduct this study. SMM (#1090440) and AGB (#1117784) are supported by National Health and Medical Research Council (NHMRC) fellowships. In addition, this study is receiving in-kind support in the form of personnel and administrative support from Metro South Health (Queensland) to enable the study to be conducted. No funding body had a role in study design, collection, management, analysis, and interpretation of data; writing of the report; and the decision to submit the report for publication.

Competing interests

None declared

Access to data

The principal investigator MRN will have full access to the final trial dataset. Associate investigators SMM, LMA, JW and AGB will have access to the final de-identified trial dataset. Other investigators will be granted access to sections of the final trial dataset. Dataset sections may include de-identified demographic data and information that pertains to data collected by the professional discipline of the associate investigator.

Data Sharing Statement

No additional data are available, though details on statistical analysis are available from the corresponding author on request.

Ancillary and post-trial care

Given the low risk of adverse events no specific provisions for ancillary and post-trial care have been made. If patients from either allocated group require follow-up services appropriate referrals for follow-up care would be made.

Ethics approval

Research Ethics Approval

Human research ethics approvals for this study have been gained from Metro South Human Research Ethics Committee (EC00167) on the 28th April 2016 (HREC/16/QPAC/193), and subsequent approval following an administrative review from Queensland University of Technology Human Research Ethics Committee (QUT reference number: 1600000441). Ethics approval has been granted until 28th April 2019.

Site specific approval (SSA) has been granted by Metro South Centres for Health Research, Research Governance (SSA/16/QPAH/195) on the 1st June 2016.

CYCLIST Study Protocol Version 2.1 dated 30th March 2017 was approved on 13th April 2017.

Participant recruitment commenced on 26th July 2016.

Provenance and peer review

Not commissioned; externally peer reviewed.

For peer review only

References:

1. Puthucheary ZA, Rawal J, McPhail M, et al. Acute Skeletal Muscle Wasting in Critical Illness. *JAMA* 2013;310(15):1591-600.

2. Gosselink R, Bott J, Johnson M, et al. Physiotherapy for adult patients with critical illness: recommendations of the European Respiratory Society and European Society of Intensive Care Medicine Task Force on Physiotherapy for Critically Ill Patients. *Intensive Care Med* 2008;34(7):1188-99.

3. Berney SC, Harrold M, Webb SA, et al. Intensive care unit mobility practices in Australia and New Zealand: a point prevalence study. *Crit Care Resusc* 2013;15(4):260-5.

4. Fan E. Critical illness neuromyopathy and the role of physical therapy and rehabilitation in critically ill patients. *Respir Care* 2012;57(6):933-44; discussion 44-6.

5. Kho ME, Martin RA, Toonstra AL, et al. Feasibility and safety of in-bed cycling for physical rehabilitation in the intensive care unit. *Journal of critical care* 2015;30(6):1419.e1-19.e5.

6. Kho ME, Molloy AJ, Clarke FJ, et al. TryCYCLE: A Prospective Study of the Safety and Feasibility of Early In-Bed Cycling in Mechanically Ventilated Patients. *PloS one* 2016;11(12):e0167561.

7. Pires-Neto CR, Fogaça Kawaguchi YM, Sayuri Hirota A, et al. Very Early Passive Cycling Exercise in Mechanically Ventilated Critically Ill Patients: Physiological and Safety Aspects - A Case Series. *PloS one* 2013;8(9):e74182.

8. Burtin C, Clerckx B, Robbeets C, et al. Early exercise in critically ill patients enhances short-term functional recovery. *Crit Care Med* 2009;37(9):2499-505.

9. Parry SM, Berney S, Warrillow S, et al. Functional electrical stimulation with cycling in the critically ill: A pilot case-matched control study. *Journal of critical care* 2014;29(4):695.e1-e7.

10. Moss M, Nordon-Craft A, Malone D, et al. A randomized trial of an intensive physical therapy program for acute respiratory failure patients. *American journal of respiratory and critical care medicine* 2016;193.

11. Hodgson CL, Iwashyna TJ, Schweickert WD. All That Work and No Gain: What Should We Do to Restore Physical Function in Our Survivors? *American journal of respiratory and critical care medicine* 2016;193(10):1071.

12. Chan A-W, Tetzlaff JM, Gøtzsche PC, et al. SPIRIT 2013 explanation and elaboration: guidance for protocols of clinical trials. *BMJ (Clinical research ed)* 2013;346(jan08 15):e7586-e86.

13. Harris PA, Taylor R, Thielke R, et al. Research electronic data capture (REDCap)—A metadata-driven methodology and workflow process for providing translational research informatics support. *Journal of Biomedical Informatics* 2009;42(2):377-81.

14. Borg G. Psychophysical scaling with applications in physical work and the perception of exertion. *Scandinavian Journal of Work, Environment & Health* 1990(1):55-58.

For peer review only

15. Berney S, Haines K, Skinner EH, et al. Safety and feasibility of an exercise prescription approach to rehabilitation across the continuum of care for survivors of critical illness. *Phys Ther* 2012;92(12):1524-35.

16. Tillquist M, Kutsogiannis DJ, Wischmeyer PE, et al. Bedside Ultrasound Is a Practical and Reliable Measurement Tool for Assessing Quadriceps Muscle Layer Thickness. *Journal of Parenteral and Enteral Nutrition* 2014;38(7):886-90.

17. De Jonghe B, Sharshar T, Lefaucheur JP, et al. Paresis acquired in the intensive care unit: a prospective multicenter study. *JAMA* 2002;288(22):2859-67.

18. Parry SM, Berney S, Granger CL, et al. A new two-tier strength assessment approach to the diagnosis of weakness in intensive care: an observational study. *Crit Care* 2015;19:52.

19. Hodgson C, Needham D, Haines K, et al. Feasibility and inter-rater reliability of the ICU Mobility Scale. *Heart Lung* 2014;43(1):19-24.

20. Huang M, Chan KS, Zanni JM, et al. Functional Status Score for the ICU: An International Clinimetric Analysis of Validity, Responsiveness, and Minimal Important Difference. *Critical Care Medicine* 2016:1.

21. Ragavan VK, Greenwood KC, Bibi K. The Functional Status Score for the Intensive Care Unit Scale: Is It Reliable in the Intensive Care Unit? Can It Be Used to Determine Discharge Placement? *Journal of Acute Care Physical Therapy* 2016;7(3):93-100.

22. ATS statement: guidelines for the six-minute walk test. *American journal of respiratory and critical care medicine* 2002;166(1):111-7.

23. Ely EW, Margolin R, Francis J, et al. Evaluation of delirium in critically ill patients: validation of the Confusion Assessment Method for the Intensive Care Unit (CAM-ICU). *Crit Care Med* 2001;29(7):1370-9.

24. Herdman M, Gudex C, Lloyd A, et al. Development and preliminary testing of the new five-level version of EQ-5D (EQ-5D-5L). *Quality of Life Research* 2011;20(10):1727-36.

25. Knaus WA, Wagner DP, Draper EA, et al. The APACHE III prognostic system. Risk prediction of hospital mortality for critically ill hospitalized adults. *Chest* 1991;100(6):1619-36.

26. Vincent JL, Moreno R, Takala J, et al. The SOFA (Sepsis-related Organ Failure Assessment) score to describe organ dysfunction/failure. On behalf of the Working Group on Sepsis-Related Problems of the European Society of Intensive Care Medicine. *Intensive Care Med* 1996;22(7):707-10.

27. Australian Code for the Responsible Conduct of Research. *Australian Government* 2007:1-41.

28. Editors. ICoMJ. Recommendations for the Conduct, Reporting, Editing and Publication of Scholarly Work in Medical Journals Available from: http://www.ICMJE.org. [accessed 01/27/2017.

For peer review only

29. Iwashyna TJ, Cooke CR, Wunsch H, et al. Population Burden of Long - Term Survivorship After Severe Sepsis in Older Americans. *Journal of the American Geriatrics Society* 2012;60(6):1070-77.

30. Herridge MS, Tansey CM, Matté A, et al. Functional disability 5 years after acute respiratory distress syndrome. *N Engl J Med* 2011;364.

31. Hodgson C, Bellomo R, Berney S, et al. Early mobilization and recovery in mechanically ventilated patients in the ICU: a bi-national, multi-centre, prospective cohort study. *Crit Care* 2015;19:81.

32. Nydahl P, Ruhl AP, Bartoszek G, et al. Early mobilization of mechanically ventilated patients: a 1-day point-prevalence study in Germany. *Crit Care Med* 2014;42.

33. Jolley SE, Moss M, Needham DM, et al. Point Prevalence Study of Mobilization Practices for Acute Respiratory Failure Patients in the United States. *Crit Care Med* 2017;45(2):205-15.

34. Elliott D, McKinley S, Alison J, et al. Health-related quality of life and physical recovery after a critical illness: a multi-centre randomised controlled trial of a home-based physical rehabilitation program. *Crit Care* 2011;15(3):R142.

For peer review only

Figure 1
Consort diagram giving flow of participants throughout the study. Abbreviations: n, number; ICU, intensive care unit.

For peer review only

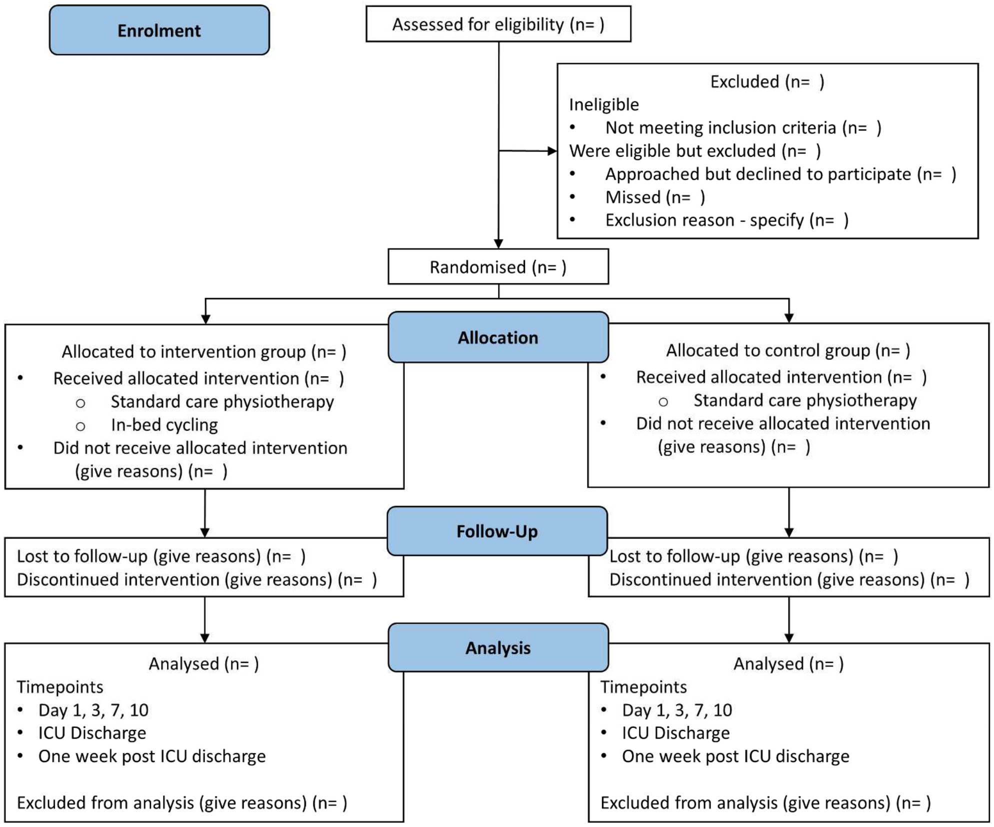

Figure 1

219x182mm (300 x 300 DPI)

Substitute Decision Maker Information Sheet

CYCLIST

Project Title	**Critical Care In-bed CYCLIng Study: A Preliminarily Randomised Controlled Trial: CYCLIST**
Site	Princess Alexandra Hospital
Principal investigator	Mr. Marc Nickels
Contact Person	Mr. Marc Nickels (07) 3176 2401, or Mrs. Chelsea Davis (07) 3176-5523
Address	Physiotherapy Department, Princess Alexandra Hospital, Ipswich Road, Woolloongabba, QLD 4102
Phone Number	07 3176 2401 or (07) 3176-5523

Participant Information Sheet

Participation in this research is voluntary. Acting on behalf of the patient, we kindly ask that you read this information sheet about this clinical trial. If you are satisfied with the explanation and agree for the patient to participate in this trial, please provide your consent on the form provided. You should keep a copy of this sheet for your and for the future reference of the patient for who you are acting on behalf of.

Introduction

This study aims to investigate whether in-bed cycling in addition to standard care reduces the rate of thigh skeletal muscle wasting and is associated with improved functional and cognitive outcomes, in critically ill patients requiring more than 48 hours of mechanical ventilation. The study will also determine if patients who participate in in-bed cycling sessions are able to walk better after discharge from intensive care compared to people who receive standard care alone. This study will also investigate which factors are linked to better outcomes.

Patients who participate in the study will be randomly allocated into either an intervention group that receives in-bed cycling sessions in addition to standard care, or into a control group that receives standard care.

Background to experiment

Current physiotherapy exercise interventions with patients in intensive care do not occur until a patient is reliably able to follow instructions. Research has shown that patients rapidly lose muscle mass whilst they are in ICU. Recent studies have also shown that cycling in-bed whilst in ICU is safe. Research has also shown that patients who complete in-bed cycling whilst in ICU have improved ability to walk at hospital discharge. Currently it is not known what the effect of in-bed cycling is on maintaining thigh muscle mass. Ultrasound is a safe way to assess patients muscle mass without any radiation exposure that occurs with other investigations such as x-rays and computerised tomography scans.

For peer review only - http://bmjopen.bmj.com/site/about/guidelines.xhtml

Description of Experiment - methods and demands

This study will test if in-bed cycling in addition to standard care affects thigh muscle loss and if there is any difference in patients' time to walk and distance walked one week after intensive care discharge.

If you chose for the patient for whom you are the substitute decision maker for to participate this means:

a) The patient will be allocated, by a random (chance) selection process, to one of the following groups:
    - Standard care (control group): Physiotherapy exercise interventions that includes sitting on the edge of the bed, moving from a bed to a chair and walking.
    - Cycling group (in-bed cycling intervention group): at least five 30 minute in-bed cycling sessions in addition to 'standard care'. In-bed cycling sessions will continue until the patient is discharged from ICU and has completed a minimum of 5 in-bed cycling sessions. The in-bed cycling sessions will continue in the acute hospital ward if the patient is discharged from ICU prior to completing 5 in-bed cycling sessions).

b) Sonographers will use ultrasound to measure the size of the patients' thigh muscles during their stay in intensive care and one week after they leave the intensive care unit.

c) Physiotherapists will measure the patients' arm, leg muscle and hand grip strength.

d) A clinical nurse will record any incidence of confusion whilst the patient is in ICU.

e) Information about the time taken for the patient to commence standing, sitting out of bed, and walking both without assistance will be recorded.

f) One week after discharge from intensive care a physiotherapist will measure how far the patient can walk in 6 minutes.

g) Information about the patient such as; gender and age, and the patients' medical condition including; diagnosis, hospital length of stay, nutritional status, that is recorded in your hospital record will be accessed by researchers to aid in result analysis.

h) You will be asked questions about your quality of life in hospital and after you return home from hospital. You may also be asked questions about your experiences with treatments you received in hospital.

i) Data collected during this study may be utilised for future research purposes following appropriate subsequent ethical review and approval.

Risk & Discomfort

Other research has established that in-bed cycling is a safe exercise for critically ill patients to participate in. It is possible that in-bed cycling may increase pain or discomfort. However, care will be taken to minimise any potential discomfort that may be experienced, and pain relief medication may be increased if deemed appropriate by the doctors. Patients will be able to request for the in-bed cycling session to be stopped.

Benefits

There may be some benefit but we don't know how much direct benefit there will be to patients participating in the study. It is possible that participation in this study will reduce patients' amount of thigh muscle wasting due to lack of use. This may correspond to a patients' improved ability to walk following a period of critical illness. We also think that participation will benefit patients, and hospitals in the future, and you and the patient may feel satisfaction at your contribution to improving health care through research.

Withdrawing from the Study

Participation is entirely voluntary and if you decide the patient will not participate in this study this will not affect the medical care or treatment of the patient (for whom you are substitute decision maker),

For peer review only - http://bmjopen.bmj.com/site/about/guidelines.xhtml

in any way. If you choose for the patient to participate, you are free to withdraw your consent and to discontinue participation of the patient at any time, by telling the research nurse. Choosing for the patient not to participate or withdrawing your consent for their participation will not affect the treatment of the patient (for whom you are the substitute decision maker) for in any way.

Confidentiality

Data collected during this study will be treated confidentially. The research nurse and assistants will store data about the patient using a unique research number. The information will be safely stored at the hospital and Queensland University of Technology. Combined patient results of this study will be published in scientific journals and presented at conferences. However, the patient will not be referred to by name and your personal identity will not be revealed in any publication or report, without specific prior approval. Research data may be accessed by auditors, the ethics committee or regulatory authorities. All research records will be confidentially destroyed 7 years after the study.
Data collected during this study may be utilised for future research purposes following appropriate subsequent ethical review and approval. If data is utilised for future research purposes, it will continue to be treated confidentially.

Contact

If you have any questions now, or at a later time, we hope and expect that you will ask us. Please contact any of the researchers named on this form by contacting *the hospital principal investigator or research nurse*, and we will be happy to answer your questions. Contact details are at the top of this form.

Metro South HHS Human Research Ethics Committee (HREC), (EC00167) and the Queensland University of Technology HREC (EC00171) have approved this study. Should you wish to discuss the study with someone not directly involved, in particular, any matters concerning policies, information about the conduct of the study or the rights of the participant, or if you wish to make a confidential complaint at any time, you may contact;

Coordinator of the Metro South HHS Human Research Ethics Committee,
Translation Research Institute, Level 7,
Woolloongabba QLD 4102
Telephone (07) 3443-8049,
email: Ethicsresearch.pah@health.qld.gov.au

For peer review only - http://bmjopen.bmj.com/site/about/guidelines.xhtml

Queensland Government

Queensland University of Technology

Participant Consent Form

HREC No:	HREC/16/QPAH/193
Project Title:	Critical Care In-bed CYCLIng STudy Preliminary Randomised Controlled Trial: CYCLIST
Name of Researchers:	Mr Marc Nickels, Senior Physiotherapist, Princess Alexandra Hospital, Tel: (07) 3176-2401; Email: marc.nickels@health.qld.gov.au
	Dr Steven McPhail, Princial Research Fellow, Centre for Functioning and Health Research, Tel: (07) 3406 2266; Email: steven.mcphail@health.qld.gov.au

Thank you for agreeing for the patient for whom you are a substitute decision maker to participate in this important research study. Although you or they may not benefit personally, you will help provide valuable information to help us to deliver safe and effective care.

I have had the contents of this information sheet explained to me and I have been provided with a copy. I agree for the patient for whom I am a substitute decision maker for to be enrolled in the project and understand that they will be randomly allocated to either a usual care group or to a usual care plus in-bed cycling group.

Please read the following carefully, and sign below if you agree with these statements and are happy to for the patient for whom you are acting as a substitute decision maker for to participate in the study:

1. I have read and understood the information sheet and this consent form.
2. I have had the opportunity to ask questions about the study and these have been answered to my satisfaction.
3. I understand that this project is for research and that participation may not have a direct benefit to the participant.
4. I have been informed that the information collected about the participant in this study will remain confidential and will be adequately safeguarded, and that when results are published, they will be presented in such a way that individuals cannot be identified.
5. I understand that if I do agree for the patient to participate, I and they are free to withdraw our consent and to discontinue participation at any time without comment, and with no effect on their treatment or relations with the Hospital in any way, but that I do need to tell the research staff if I wish to withdraw the patient for whom I am a substitute decision maker for.
6. If I have any questions or comments about the study at any time I am free to contact Mr Marc Nickels on (07) 3176-2401 or the research nurse on (07) 3176 5523.
7. If I have any complaints about the ethical conduct of the study, I may direct these to the, Coordinator of the Ethics Committee, Princess Alexandra Hospital, on (07) 3176-8049.

For peer review only - http://bmjopen.bmj.com/site/about/guidelines.xhtml

I agree for the patient for whom I am a substitute decision maker for, to participate in the study and I give permission for authorised study personnel to extract details that pertain to this study from the patients' hospital medical record.

Name: ……………………………………….Signature………………………….. Date: __/__/__

Witness: .... Signature………………………….. Date: __/__/__

Enrolled by: .... Signature………………………….. Date: __/__/__

For peer review only

For peer review only - http://bmjopen.bmj.com/site/about/guidelines.xhtml

Queensland Government

Queensland University of Technology

Revocation of Consent Form - Participant

HREC No:	HREC/16/QPAH/193
Project Title:	Critical Care In-bed CYCLIng STudy Preliminary Randomised Controlled Trial: CYCLIST
Name of Researchers:	Mr Marc Nickels, Senior Physiotherapist, Princess Alexandra Hospital, Tel: (07) 3176-2401; Email: marc.nickels@health.qld.gov.au
	Dr Steven McPhail, Princial Research Fellow, Centre for Functioning and Health Research, Tel: (07) 3406 2266; Email: steven.mcphail@health.qld.gov.au

(TO BE USED FOR PARTICIPANTS WHO WISH TO WITHDRAW FROM THE PROJECT)

- I hereby wish to WITHDRAW my consent for the patient for whom I am a substitute decision maker for to participate in the research proposal described above.
- I understand that such withdrawal WILL NOT jeopardise any treatment of the patient for whom I am a substitute decision maker for or relationship with the Princess Alexandra Hospital or Queensland University of Technology.

Participant's Name (printed)

____

________________________________ __________________

Signature Date

Please send to:
Clinical Research Nurse
Intensive Care Unit
Princess Alexandra Hospital
199 Ipswich Rd
Woolloongabba QLD 4102

For peer review only - http://bmjopen.bmj.com/site/about/guidelines.xhtml

SPIRIT

STANDARD PROTOCOL ITEMS: RECOMMENDATIONS FOR INTERVENTIONAL TRIALS

SPIRIT 2013 Checklist: Recommended items to address in a clinical trial protocol and related documents*

Section/item	Item No	Description	Location in manuscript
**Administrative information**			
Title	1	Descriptive title identifying the study design, population, interventions, and, if applicable, trial acronym	Title, page 1
Trial registration	2a	Trial identifier and registry name. If not yet registered, name of intended registry	Abstract, page 2
	2b	All items from the World Health Organization Trial Registration Data Set	Throughout manuscript
Protocol version	3	Date and version identifier	Additional information, page 17
Funding	4	Sources and types of financial, material, and other support	Additional information, page 16
Roles and responsibilities	5a	Names, affiliations, and roles of protocol contributors	Additional information, page 16
	5b	Name and contact information for the trial sponsor	Additional information page 16
	5c	Role of study sponsor and funders, if any, in study design; collection, management, analysis, and interpretation of data; writing of the report; and the decision to submit the report for publication, including whether they will have ultimate authority over any of these activities	Additional information, page 16

For peer review only

	5d	Composition, roles, and responsibilities of the coordinating centre, steering committee, endpoint adjudication committee, data management team, and other individuals or groups overseeing the trial, if applicable (see Item 21a for data monitoring committee)	Trial Management, page 13
**Introduction**			
Background and rationale	6a	Description of research question and justification for undertaking the trial, including summary of relevant studies (published and unpublished) examining benefits and harms for each intervention	Introduction, page 3
	6b	Explanation for choice of comparators	Introduction, page 3
Objectives	7	Specific objectives or hypotheses	Objectives, page 5
Trial design	8	Description of trial design including type of trial (eg, parallel group, crossover, factorial, single group), allocation ratio, and framework (eg, superiority, equivalence, noninferiority, exploratory)	Methods, Study Design and Setting, page 5
**Methods: Participants, interventions, and outcomes**			
Study setting	9	Description of study settings (eg, community clinic, academic hospital) and list of countries where data will be collected. Reference to where list of study sites can be obtained	Methods, Study Design and Setting, page 5
Eligibility criteria	10	Inclusion and exclusion criteria for participants. If applicable, eligibility criteria for study centres and individuals who will perform the interventions (eg, surgeons, psychotherapists)	Table 1, Inclusion and exclusion criteria, page 6
Interventions	11a	Interventions for each group with sufficient detail to allow replication, including how and when they will be administered	Study Intervention, page 7-8

For peer review only

	11b	Criteria for discontinuing or modifying allocated interventions for a given trial participant (eg, drug dose change in response to harms, participant request, or improving/worsening disease)	Table 2, Safety Guidelines, page 8
	11c	Strategies to improve adherence to intervention protocols, and any procedures for monitoring adherence (eg, drug tablet return, laboratory tests)	Study Intervention, page 8
	11d	Relevant concomitant care and interventions that are permitted or prohibited during the trial	Study Intervention, page 8
Outcomes	12	Primary, secondary, and other outcomes, including the specific measurement variable (eg, systolic blood pressure), analysis metric (eg, change from baseline, final value, time to event), method of aggregation (eg, median, proportion), and time point for each outcome. Explanation of the clinical relevance of chosen efficacy and harm outcomes is strongly recommended	Outcomes, page 9<br>Table 3
Participant timeline	13	Time schedule of enrolment, interventions (including any run-ins and washouts), assessments, and visits for participants. A schematic diagram is highly recommended (see Figure)	Figure 1
Sample size	14	Estimated number of participants needed to achieve study objectives and how it was determined, including clinical and statistical assumptions supporting any sample size calculations	Sample size, page 10
Recruitment	15	Strategies for achieving adequate participant enrolment to reach target sample size	Trial Management page 13.
**Methods: Assignment of interventions (for controlled trials)**			

Allocation:			
Sequence generation	16a	Method of generating the allocation sequence (eg, computer-generated random numbers), and list of any factors for stratification. To reduce predictability of a random sequence, details of any planned restriction (eg, blocking) should be provided in a separate document that is unavailable to those who enrol participants or assign interventions	Randomisation, page 6
Allocation concealment mechanism	16b	Mechanism of implementing the allocation sequence (eg, central telephone; sequentially numbered, opaque, sealed envelopes), describing any steps to conceal the sequence until interventions are assigned	Randomisation, page 6
Implementation	16c	Who will generate the allocation sequence, who will enrol participants, and who will assign participants to interventions	Randomisation, page 6
Blinding (masking)	17a	Who will be blinded after assignment to interventions (eg, trial participants, care providers, outcome assessors, data analysts), and how	Data collection, page 12
	17b	If blinded, circumstances under which unblinding is permissible, and procedure for revealing a participant's allocated intervention during the trial	Data collection, page 12
**Methods: Data collection, management, and analysis**			

Data collection methods	18a	Plans for assessment and collection of outcome, baseline, and other trial data, including any related processes to promote data quality (eg, duplicate measurements, training of assessors) and a description of study instruments (eg, questionnaires, laboratory tests) along with their reliability and validity, if known. Reference to where data collection forms can be found, if not in the protocol	Data collection, page 10-12
	18b	Plans to promote participant retention and complete follow-up, including list of any outcome data to be collected for participants who discontinue or deviate from intervention protocols	Study Intervention, page 8
Data management	19	Plans for data entry, coding, security, and storage, including any related processes to promote data quality (eg, double data entry; range checks for data values). Reference to where details of data management procedures can be found, if not in the protocol	Data collection, page 10-12
Statistical methods	20a	Statistical methods for analysing primary and secondary outcomes. Reference to where other details of the statistical analysis plan can be found, if not in the protocol	Analysis, page 12-13
	20b	Methods for any additional analyses (eg, subgroup and adjusted analyses)	Analysis, Table 5, page 13
	20c	Definition of analysis population relating to protocol non-adherence (eg, as randomised analysis), and any statistical methods to handle missing data (eg, multiple imputation)	Analysis, page 12-13
**Methods: Monitoring**			

Data monitoring	21a	Composition of data monitoring committee (DMC); summary of its role and reporting structure; statement of whether it is independent from the sponsor and competing interests; and reference to where further details about its charter can be found, if not in the protocol. Alternatively, an explanation of why a DMC is not needed	Contributors, page 16
	21b	Description of any interim analyses and stopping guidelines, including who will have access to these interim results and make the final decision to terminate the trial	Trial management, page 13
Harms	22	Plans for collecting, assessing, reporting, and managing solicited and spontaneously reported adverse events and other unintended effects of trial interventions or trial conduct	Trial management, page 13
Auditing	23	Frequency and procedures for auditing trial conduct, if any, and whether the process will be independent from investigators and the sponsor	Trial management, page 13
**Ethics and dissemination**			
Research ethics approval	24	Plans for seeking research ethics committee/institutional review board (REC/IRB) approval	Abstract, page 2
Protocol amendments	25	Plans for communicating important protocol modifications (eg, changes to eligibility criteria, outcomes, analyses) to relevant parties (eg, investigators, REC/IRBs, trial participants, trial registries, journals, regulators)	Analysis, page 12
Consent or assent	26a	Who will obtain informed consent or assent from potential trial participants or authorised surrogates, and how (see Item 32)	Consent, page 5-6

w only

	26b	Additional consent provisions for collection and use of participant data and biological specimens in ancillary studies, if applicable	Not applicable
Confidentiality	27	How personal information about potential and enrolled participants will be collected, shared, and maintained in order to protect confidentiality before, during, and after the trial	Data Collection, page 11
Declaration of interests	28	Financial and other competing interests for principal investigators for the overall trial and each study site	Additional information, page 16
Access to data	29	Statement of who will have access to the final trial dataset, and disclosure of contractual agreements that limit such access for investigators	Additional information, page 16
Ancillary and post-trial care	30	Provisions, if any, for ancillary and post-trial care, and for compensation to those who suffer harm from trial participation	Additional information, page 16
Dissemination policy	31a	Plans for investigators and sponsor to communicate trial results to participants, healthcare professionals, the public, and other relevant groups (eg, via publication, reporting in results databases, or other data sharing arrangements), including any publication restrictions	Dissemination, page 13
	31b	Authorship eligibility guidelines and any intended use of professional writers	Dissemination, page 13
	31c	Plans, if any, for granting public access to the full protocol, participant-level dataset, and statistical code	Dissemination, page 13
**Appendices**			

For peer review only

Informed consent materials	32	Model consent form and other related documentation given to participants and authorised surrogates	Substitute Decision Maker Consent Form
Biological specimens	33	Plans for collection, laboratory evaluation, and storage of biological specimens for genetic or molecular analysis in the current trial and for future use in ancillary studies, if applicable	Not applicable

*It is strongly recommended that this checklist be read in conjunction with the SPIRIT 2013 Explanation & Elaboration for important clarification on the items. Amendments to the protocol should be tracked and dated. The SPIRIT checklist is copyrighted by the SPIRIT Group under the Creative Commons "Attribution-NonCommercial-NoDerivs 3.0 Unported" license.

For peer review only

BMJ Open

Critical Care Cycling Study (CYCLIST) Trial Protocol: a randomised controlled trial of usual care versus usual care plus additional in-bed cycling sessions in the critically ill

Journal:	*BMJ Open*
Manuscript ID	bmjopen-2017-017393.R2
Article Type:	Protocol
Date Submitted by the Author:	09-Aug-2017
Complete List of Authors:	Nickels, Marc; Princess Alexandra Hospital, Physiotherapy Department<br>Aitken, Leanne M.; City, University of London, School of Health Sciences; Griffith University Menzies Health Institute Queensland, National Centre of Research Excellence in Nursing Interventions for Hospitalised Patients<br>Walsham, James; Princess Alexandra Hospital, Intensive Care Unit; University of Queensland<br>Barnett, Adrian; Queensland University of Technology, Institute of Health and Biomedical Innovation<br>McPhail, Steven; Queensland University of Technology, Institute of Health and Biomedical Innovation; Metro South Hospital and Health Service, Centre for Functioning and Health Research
**Primary Subject Heading**:	Intensive care
Secondary Subject Heading:	Rehabilitation medicine
Keywords:	INTENSIVE & CRITICAL CARE, REHABILITATION MEDICINE, cycle ergometry, muscle atrophy, physiotherapy, critical illness

For peer review only

SCHOLARONE™
Manuscripts

Critical Care Cycling Study (CYCLIST) Trial Protocol: a randomised controlled trial of usual care versus usual care plus additional in-bed cycling sessions in the critically ill

Authors

1. Marc R Nickels [a, *]
2. Leanne M Aitken [b, c, d]
3. James Walsham [d, e]
4. Adrian G Barnett [f]
5. Steven M McPhail [f, g]

a) Intensive Care Unit and Physiotherapy Department, Princess Alexandra Hospital, Brisbane, Australia

b) School of Health Sciences, City, University of London, London, United Kingdom

c) National Centre of Research Excellence in Nursing Interventions for Hospitalised Patients, Menzies Health Institute Queensland, Griffith University, Brisbane, Australia

d) Intensive Care Unit, Princess Alexandra Hospital, Brisbane, Australia

e) University of Queensland, Brisbane, Australia

f) School of Public Health & Social Work and Institute of Health and Biomedical Innovation, Queensland University of Technology, Brisbane, Australia

g) Centre for Functioning and Health Research, Metro South, Brisbane, Australia

* Corresponding author at: Intensive Care Unit and Physiotherapy Department, Princess Alexandra Hospital, Brisbane, Australia, 4102. Tel.: +61 7 3176 2401. E-mail address: marc.nickels@health.qld.gov.au (M. Nickels).

Keywords:

Critical illness; Cycle ergometry; Intensive care units; Muscle weakness; Rehabilitation

For peer review only

ABSTRACT

Introduction

In-bed cycling with critically ill patients has been shown to be safe, feasible and improve physical function outcomes at hospital discharge. The effect of early in-bed cycling on reducing the rate of skeletal muscle atrophy, and associations with physical and cognitive function are unknown.

Methods and analysis

A single centre randomised controlled trial in a mixed medical-surgical intensive care unit (ICU) will be conducted. Adult patients (n= 68) who are expected to be mechanically ventilated for more than 48 hours and remain in ICU for a further 48 hours from recruitment will be randomly allocated into either (1) a usual care group or (2) a group that receives usual care and additional in-bed cycling sessions. The primary outcome is change in rectus femoris cross-sectional area at day 10 in comparison to baseline measured by blinded assessors. Secondary outcome measures include muscle strength, incidence of ICU acquired weakness, handgrip strength, time to achieve functional milestones (sitting out of bed, walking), Functional Status Score in ICU, ICU mobility scale, six-minute walk test one week post ICU discharge, incidence of delirium and quality of life (EQ-5D-5L). Quality of life assessments will be conducted at day 10 post ICU admission and 3 and 6 months post hospital discharge. Participants in the intervention group will complete an acceptability of intervention questionnaire.

Ethics and dissemination

Appropriate ethical approval from Metro South Health Human Research Ethics Committee has been attained. Results will be published in peer-reviewed publications and presented at scientific conferences to assist planning of future multi-centre randomised controlled trials (if indicated) that will test in-bed cycling as an intervention to improve the physical, cognitive and health-related quality of life outcomes of critically ill patients.

Trial Registration

This trial has been prospectively registered on the Australian and New Zealand Clinical Trial Registry (ACTRN12616000948493).

For peer review only

Strengths and Limitiations of this study

- The randomised trial design with blinded assessments of skeletal muscle size, strength and function to provide objective measures of difference
- The inclusion of an acceptability questionnaire will provide useful insights for subsequent implementation (if indicated)
- The study may not be powered for all secondary outcomes, and evidence of effect size from pilot data is not available for those measures

For peer review only

Background and Rationale

Critically ill patients often require mechanical ventilation for periods greater than 48 hours. It has been identified that skeletal muscle wasting occurs early and rapidly during the first week of critical illness [1]. Despite international recommendations for critically ill patients to commence activity as early as possible [2] it has been identified that exercise interventions are rarely initiated when a patient is on mechanical ventilation [3]. This leads to prolonged immobility and may contribute to the development of intensive care unit acquired weakness (ICUAW) [4]. In-bed cycling using a cycle ergometer has been proposed as a safe and feasible method of introducing early exercise for critically ill patients on mechanical ventilation who are sedated and immobile, this includes patients requiring inotropic support [5-7]. In-bed cycling may also assist in the preservation of muscle architecture. To date only one randomised controlled trial (RCT) utilising in-bed cycling in the critically ill population has been published [8]. This single centre RCT (n = 90) conducted in Belgium, found in-bed cycling to be safe and to improve critically ill patients' 6-minute walk distance, quadriceps force and Short Form-36 (SF-36) physical function scores on hospital discharge [8]. A limitation of this study was that the effects of the intervention at the muscular level were not assessed with muscle biopsy or ultrasound.[8] A pilot case-matched study of in-bed Functional Electrical Stimulated (FES) cycling intervention in addition to usual care, found positive physical outcomes observed among the cycling group including less time required to achieve functional milestones, time to stand and time to ambulate independently [9]. In addition, a shorter duration of delirium among those who participated in the Functional Electrical Stimulation in-bed cycling intervention was observed [9].

A recent clinical trial has demonstrated that critically ill patients may experience persistent weakness despite participating in intensive exercise programs whilst they are critically ill [10]. It has been suggested that intensive exercise programs may not be effective if the commencement of these programs is delayed [11]. Early exercise commencement is intended to assist in the maintenance of muscle mass. This may be achieved through a moderation of the inflammatory process [11]. Consequently, the effectiveness of exercise interventions that can commence early during critical illness are necessary to demonstrate if patient outcomes are improved by the early commencement of exercise during a period of critical illness.

Early clinical studies in the field have demonstrated potential for in-bed cycling interventions (with and without Functional Electrical Simulation) to improve physical and cognitive function among critically ill patients [8 9]. There are currently no published RCTs investigating the effectiveness of in-bed cycling in critically ill patients requiring prolonged mechanical ventilation on quadriceps structure, ICU-acquired weakness and cognitive outcomes. Consequently, further investigation of

For peer review only

the effect of in-bed cycling on muscle structure, physical function and cognitive function is warranted.

Objectives

The objectives of this study are to:

(1) Examine whether in-bed cycling in addition to usual care is effective in reducing the rate of rectus femoris cross-sectional area (CSA) atrophy and ICUAW in patients requiring more than 48 hours of invasive mechanical ventilation compared with usual care.

(2) Investigate if in-bed cycling in addition to usual care is associated with better functional and cognitive outcomes in patients predicted to require more than 48 hours of invasive mechanical ventilation compared to usual care.

METHODS

Study Design and Setting

This trial will be a two arm, parallel randomised controlled trial with individual participant allocation and blinding of the primary outcome assessor. It will be conducted in a 25-bed tertiary mixed medical and surgical adult intensive care unit in Brisbane, Australia. Participants will be allocated 1:1 to receive either usual care or in-bed cycling in addition to usual care (Figure 1). In designing this study, the SPIRIT 2013 Checklist was utilised to ensure that all recommended items in a clinical trial were addressed [12].

Consent

It is anticipated that most patients who are eligible to participate in this study in the ICU setting will not be able to provide informed consent at the time of study enrolment. For those patients that may have the capacity to provide informed consent, the Richmond Agitation and Sedation Scale (RASS) will be used to determine if a patient is rated 'Alert and Calm'. If a patient is rated as 'Alert and Calm' on the RASS, the Confusion Assessment Method for the ICU (CAM-ICU) will be used to determine if a patient has had delirium within the preceding 24 hours. Provided a patient passes the RASS and CAM-ICU assessments the treating clinical team will be approached to determine if the patient has the capacity to provide informed consent. Patients without delirium for the preceding 24 hours and deemed to have capacity will be approached to provide their own written informed consent for study participation. For eligible patients considered unable to provide informed consent at the time of study enrolment, substitute decision makers (family members or next of kin), will be approached for written informed consent. The Queensland Civil and Administrative Tribunal (QCAT) have approved an application to provide consent for individuals who are unable to give consent and do

For peer review only

not have a next of kin that is accessible to request consent on the patients' behalf. Delayed consent from the patient will be sought once they can provide consent for themselves if they are enrolled using QCAT approval. Participation in the study is voluntary. Patients or their substitute decision makers are able to withdraw at any time without any negative consequences on the care they would receive, their ongoing relationship with the hospital, or staff involved in their care.

The substitute decision maker consent form (PICF_SDM_CYCLIST_V3.0_20160701) is attached as a supplementary file.

Randomisation

Patients will be individually randomised in a 1:1 ratio to either intervention or usual care group. Blocking (random block sizes) will be used to help balance the groups. An investigator not involved in the screening, consenting, allocation or assessment processes will use computerised random number generation to create the randomisation sequence. A randomisation sequence will be uploaded onto the Research Electronic Data Capture (REDCap) secure web based computer application [13]. The REDCap randomisation module will reveal the group allocation of each patient to the intervention coordinator after a patients' baseline data has been collected.

Participants

The study aims to recruit 68 participants. Adult patients expected to require at least 48 hours of mechanical ventilation will be recruited, with the inclusion and exclusion criteria listed in Table 1. Criteria to guide when to discontinue or not deliver an intervention are listed in Table 2. Participants can be recruited into the study and baseline sonography measures performed if a patient does not curently meet the in-bed cycling safety criteria in Table 2 (but are considered by their treating clinical team to be likely to meet the criteria within their stay in ICU).

Table 1 Inclusion and exclusion criteria

Inclusion	Exclusion
Expected to require more than 48 hours of invasive mechanical ventilation	Pre-existing condition that is likely to impair mobility/ mobility assessment (i.e. significant neurological, musculoskeletal, cognitive or mental health disorder)
Able to provide consent or have a family member consent on their behalf	Neuromuscular disorder or acute primary brain lesion (i.e. traumatic brain injury, intracranial haemorrhage, stroke, or hypoxic brain injury)
Enrolled into the study within 96 hours of ICU admission	Injuries precluding cycle ergometry (i.e. spinal / pelvic / lower limb orthopaedic injuries / open abdominal wound)
Expected to remain in ICU for more than 48 hours following study enrolment	Obesity > 135 kilograms (MOTOmed Letto 2 maximum rated weight capacity)
	Uncontrolled seizures or status epilepticus
	Dire prognosis (i.e. unlikely to survive the current admission)

For peer review only

	Pregnancy
	Children and/or young people (i.e. < 18 years)

Study Intervention

All study patients will receive usual physiotherapy interventions whilst in intensive care. Physiotherapy interventions will include (but are not limited to): respiratory physiotherapy, physical rehabilitation exercise interventions including sitting on the edge of the bed, sit to stand transfers, sitting out of bed and walking. Usual care physiotherapy interventions will be prioritised over the in-bed cycling intervention. Safety guidelines (see Table 2) will be used to determine if the intervention group patients are able to complete an additional daily 30-minute progressive lower limb in-bed cycling using a bedside cycle ergometer (MOTOmed Letto 2). The lead physiotherapist (MRN) who has over 10 years of experience in rehabilitative exercise with critically ill patients will primarily conduct the majority of in-bed cycling sessions. He has been trained by industry cycle ergometry representatives and has over 5 years of experience conducting in-bed cycling sessions with critically ill patients. Experienced ICU physiotherapists trained in conducting in-bed cycling sessions may conduct the in-bed cycling sessions if the lead physiotherapist is unavailable. During the in-bed cycling exercise interventions the patients' vital signs will be monitored. If the intervention group are in a state of low arousal or sedated they will cycle continuously and passively at the default passive speed of the cycle ergometer (20 revolutions per minute) for 30 minutes. When the participant is following commands, the clinician will verbally encourage the patient to complete in-bed cycling sessions actively for a duration of 30 minutes. Once the patient can cycle actively the resistance applied by the cycle ergometer will be adjusted to facilitate patient intensity of between 3 and 5 using the visual Borg scale rate of perceived exertion (category ratio 10),[14] within the specified safety guidelines. An exercise intensity of 3-5 on the Borg rate of perceived exertion scale has been shown to be safe and feasible with critically ill patients.[15] This will enable the patient to cycle in-bed either passively or actively with assistance from the cycle ergometer. If a patient unexpectedly commences active cycling the additional active in-bed cycling safety criteria will become relevant. If the patient is deemed unsuitable to continue active in-bed cycling they will be asked to resume passive cycling. If the patient continues to actively cycle the session will be ceased. The in-bed cycling sessions will continue until the patient completes a minimum of 5 in-bed cycling sessions, unless the patient is discharged from hospital prior to completing 5 sessions. The intervention will continue in the acute hospital ward if the patient is discharged from ICU prior to completing 5 in-bed cycling sessions. Whilst the patient remains in ICU, in-bed cycling sessions will continue (up to 7 days per week), up to 28 days' post ICU admission. This frequency is congruent with usual physiotherapy services that can provide rehabilitative exercise interventions to ICU patients up to seven days per week. Patients

For peer review only

randomised to the usual care arm do not routinely complete in-bed cycling sessions during their hospitalisation. Any deviations from the planned protocol will be recorded to enable appropriate intervention description and if indicated a per-protocol analysis (in addition to the primary intention to treat analysis).

Participants will not be coerced to complete any intervention or outcome measure. Participants' may be discharged home from the participating acute hospital before they have completed outcome measures at each assessment time-point. If this occurs, participants will be asked to return to the hospital to enable the remaining outcome measures to be completed and expenses related to taxi or parking costs will be reimbursed.

Table 2 Safety guidelines

**Active or passive exercise should not be delivered if:**
Clinician opinion that patient condition unstable
Resting HR < 40 or > 120 bpm or new arrhythmia
Evidence of coronary ischaemia e.g. chest pain or ECG changes
MAP < 60 or SBP > 200 mmHg
SpO2 < 90%
RASS ≥ 2
Wounds of leg, pelvis or lumbar spine precluding cycle ergometry
Evidence of active bleeding or coagulation disorder: INR >1.8, PLT <50,000/microL. *
Femoral vascular access ** e.g. dialysis catheter, IABP, ECMO or lower limb arterial line ***
Acute DVT or PE
**Active exercise should not be delivered if:**
> 20 µg/min of noradrenaline or comparable inotropic or vasopressor support
FiO2 > 0.55 or PEEP > 10 cmH2O
RR > 30 with adequate ventilatory support
Temperature > 39° Celsius
**Stopping criteria: active or passive exercise should cease if:**
HR < 50 or > 140 bpm, or new arrhythmia develops (including ventricular ectopic or new-onset AF)
Evidence of coronary ischaemia e.g. chest pain or ECG changes
MAP < 60 mmHg
SBP > 200 mmHg
Clinical signs of cardiorespiratory distress
SpO2 < 90% for more than 1 minute
Patient request to stop therapy

HR, heart rate; bpm, beats per minute; ECG, electrocardiogram; FIO2, fraction of inspired oxygen; MAP, mean arterial pressure; SBP, systolic blood pressure; DBP, diastolic blood pressure; SpO2, saturation of peripheral oxygen; RASS, Richmond Agitation Sedation Score; INR, international normalised ratio; PLT, platelets; microL, microlitre; IABP, intra-aortic balloon pump; ECMO, extracorporeal membrane oxygenation; DVT, deep vein thrombosis; PE, pulmonary embolism; PEEP, positive end expiratory pressure; RR, respiratory rate; AF, atrial fibrillation

** Values outside this range would be tolerated if patient therapeutically anticoagulated*

*** Other than femoral central line*

****If a femoral vascular access is inserted unilaterally the contralateral leg may be cycled unilaterally.*

Outcomes

Table 3 provides a summary of the outcome measures. The primary outcome is the percentage of change in rectus femoris CSA measured at baseline (within 24 hours of study enrolment) and day 10 (post study enrolment), measured by a blinded assessor. The participants will be assessed to examine whether there is a between group difference in the amount of rectus femoris CSA atrophy. The authors anticipate that less rectus femoris CSA atrophy will be observed among the in-bed cycling group. Day 10 post study enrolment has been chosen as the primary endpoint to enable comparison with previously published data on acute skeletal muscle wasting in patients with critical illness and consistent with this previously reported time-frame of observed muscle wasting.[1] Secondary outcome measures in the CYCLIST study are muscle strength, physical function, cognition, quality of life, and acceptability of intervention.

Table 3 Descriptions of outcome measures for CYCLIST RCT

Assessment Component	Outcome Measure	Description
Muscle Morphology	Ultrasound	RF CSA, AP thickness of RF and VI. Measured in triplicate on right anterior thigh one third distance from superior patella to ASIS Patient positioned in supine, thirty degress head elevation [16]
Muscle Strength	MRC Sum Score	Standardised sum of twelve MMTs, three MMTs per limb Score ≤ 48 indicative of ICU acquired weakness [17]
	Handgrip strength dynamometry	Triplicate bilateral measurement using a Jamar Digital Dynamometer (Lafayette) with seated patient [18]
Physical Function	ICU Mobility Scale	Best level of function achieved in ICU using an eleven-point ordinal scale [19]
	FSS-ICU	Patients' function measured an eight-point ordinal scale [20 21]
	Functional milestones	Time to achieve functional milestones: Sit out of bed, time to stand, mobilise with assistance and mobilise independently
	6-minute walk test	Sub-maximal endurance test of distance walked by a patient in six minutes [22]
Cognition	CAM-ICU	Incidence and recorded episodes of acute delirium [23]
Quality of Life	EQ-5D-5L	EuroQol five dimensions questionnaire five level scale [24]
Intervention Acceptability	Customised questionnaires	Questionnaire about the acceptability of the in-bed cycling intervention

RF, rectus femoris; CSA, cross sectional area; VI, vastus intermedius; AP, anterior posterior; ASIS, anterior superior illiac crest; MRC, Medical Research Council; MMT, manual muscle test; ICU, intensive care unit; FSS-ICU, Funcitonal Status Score for the Intensive Care Unit; CAM-ICU Confusion Assessment Measure for the Intensive Care Unit; EQ-5D-5L, EuroQol five dimensions questionnaire five level scale.

Demographic information such as age, gender and diagnostic code will be collected. Illness related information including length of mechanical ventilation, ICU (LOS) and hospital LOS and discharge destination, illness severity (APACHE III [25], Sequential Organ Failure Assessment (SOFA) [26] score), pre-

For peer review only

morbid co-morbidities, neuromuscular blockade and sedation medications administered, nutrition received, cumulative fluid balance, patient height and weight, and body mass index will also be collected.

Sample Size

Based on the repeated measures design this study will have 80% power to detect a between groups difference of 2.9% on our primary outcome (change in rectus femoris CSA) between the intervention and usual care participants, (assuming type I error 0.05, standard deviation of 6%, and within-patient correlation of 0.5 between assessments, and after accounting for an up to 20% drop out rate including in-hospital mortality). A total of 68 patients (34 in each group) will be recruited into the study. With a drop-out rate of up to 20%, this would represent approximately 27 participants completing the trial in each group. It is noteworthy that some of the assumptions for this calculation have been made in the absence of prior randomised control trial data. There is some risk that the observed trial data may not fit these assumptions (e.g., discrepancies in standard deviations or within-patient correlation between the repeated measures), and the power of the study may differ from this a-priori sample size estimate.

Data Collection

The study data will be collected at the time points summarised in Table 4.

Table 4 CYCLIST summary of timepoints of assessments

	Baseline	Day 3	Day 7	Day 10	ICU discharge	1 week post ICU discharge*	3, 6 months' post hospital discharge
Severity of illness							
APACHE III	✓						
SOFA	✓	✓	✓	✓	✓		
Muscle Morphology							
Quadriceps Ultrasonography	✓	✓	✓	✓		✓	
Strength Measures							
MRC Sum Score					✓	✓	
Handgrip dynamometry					✓	✓	
Physical Function Measures							
ICU Mobility Scale					✓		
FSS-ICU					✓		
Functional Milestones	✓	✓	✓	✓	✓	✓	
6-MWT						✓	
Cognition							
CAM-ICU	✓	✓	✓	✓	✓		
Quality of Life							
EQ-5D-5L				✓			✓

For peer review only - http://bmjopen.bmj.com/site/about/guidelines.xhtml

**Acceptability of Intervention**			
	✓**		
**Patient Outcomes**			
ICU Length of Stay	✓		
Hospital Length of Stay		✓***	
Acute discharge destination		✓***	
Mortality	✓	✓***	✓

ICU, intensive care unit; APACHE, Acute Physiology and Chronic Health Evaluation; SOFA, Sequential Organ Failure Assessment score; MRC, Medical Research Council; FSS-ICU, functional status score in the intensive care unit; CAM-ICU, confusion assessment measure in intensive care; EQ-5D-5L, EuroQol five dimensions. questionnaire five level scale.

** 1 week post ICU discharge or at acute hospital discharge if sooner.*

*** At completion of in-bed cycle ergometry sessions*

**** Measured at acute hospital discharge*

For peer review only

The management of study data will be dependent on the type of data collected. Baseline data will be entered directly into Research Electronic Data Capture (REDCap) designed digital clinical trial workflow management software [13]. Ultrasound results will be uploaded onto the secure hospital based AGFA IMPAX 6.5.3.1005 Medical Image Viewer application. In-bed cycling session data, physical assessment measures, acceptability of in-bed cycling intervention and quality of life questionnaires will be recorded initially onto research data sheets. All information recorded on data sheets will be subsequently entered into the REDCap application.

Assessments of muscle size will be assessed by ultrasound and performed by registered post-graduate trained sonographers with expertise in musculoskeletal sonography. Sonographers' will be blinded to patient treatment group allocation and will be responsible for analysing and scoring all ultrasound images. The study sonographers were involved in the development and standardisation of the ultrasound procedure prior to the commencement of the study.

Assessment of muscle strength and function will be completed by cardiorespiratory physiotherapy assessors with a minimum of 3 years' experience at participants' ICU discharge and 7-days post ICU discharge. These physiotherapists will be blinded to group allocation. The same physiotherapists will also assess 6-minute walk test distance 7-days following ICU discharge. The study physiotherapists were trained in the standardised assessment of the study outcome measures prior to the commencement of the study.

A physiotherapist not involved in blinded outcome assessment will be responsible for conducting the in-bed cycling sessions, and the remaining 'usual care' physiotherapy will be completed by hospital

department physiotherapists not involved in the study. Additionally, the primary statistician will be blinded to treatment group allocation.

To minimise the chances of unintentional unblinding, at the beginning of each assessment the blinded assessors will clearly state that they wish to complete the assessment process without knowing which group the patient was allocated too. If any blinded assessors were to become unblinded to group allocation for any patient (e.g., through inadvertent revelation by a patient being assessed or unanticipated exposure to a patient taking part in an in-bed cycling session), they have been instructed to report this to the intervention coordinator (MRN) who will also record when this occurred.

ANALYSIS

Data will be analysed and reported using intention to treat principles (primary analysis). A per protocol analysis will be conducted to assist in determining the efficacy of the in-bed cycling protocol if variation from the planned protocol occurs for a substantial proportion of patients. The per protocol analysis will include only patients who adhered to the protocol and received at least 80% of training sessions (minimum of 4 sessions).

Descriptive statistics and generalized linear mixed models (that can adjust for baseline status) will be used to examine the effect of group allocation (intervention vs. usual care) on the primary and secondary outcomes [27]. As this is a randomised trial we do not plan to adjust for potential confounders (e.g., age, gender, comorbidities), but will compare the characteristics of the sample by treatment group and may adjust if a potential confounder differs greatly between groups.

If blinded sonographers are unable to complete an ultrasound measurement at designated time-points, then the scan will be performed within a day (± 1 day) of the designated time-point. A subsequent sensitivity analysis will be conducted to determine if there is a difference by measurement timing. If the primary outcome cannot be collected by a sonographer within the specified timeframe a physiotherapist experienced in musculoskeletal ultrasound will measure thigh muscle size. The measurement will be from the most anterior aspect of rectus femoris to the anterior surface of the femur. This will assist in the imputation of the missing primary outcome data. Statistical analysis of available data will be used for the primary analysis when data is completely missing. Multiple imputation will be conducted if more than 20% of outcome data is missing. Additional planned secondary analyses are listed in Table 5. The principal investigators will

For peer review only

determine if study protocol modifications are required and any modifications to the existing protocol will be declared.

Table 5 Planned secondary analyses

Muscle wasting at baseline, day 3, day 7 and 1 week post ICU discharge
Muscle wasting adjusted for number of failing vital organs *
Muscle wasting adjusted for severity of illness on admission to ICU **
Muscle wasting adjusted for the number of days prior to a patient commencing active activity
Muscle wasting adjusted for the patients' cumulative fluid balance on the day of the ultrasound scan
Relationship between muscle wasting and participants' nutritional intake whilst in ICU
Relationship between sedative and paralytic medications and muscle wasting
Cost comparision of hospitalisation for both the intervention and usual care groups ***
Hospital readmissions post acute hospital discharge over a two year time period ****
Mortality *****

Utilising: * Sequential Organ Failure Score (SOFA), ** APACHE III data, *** health service utilisation data, **** Queensland Hospital Admitted Patient Data Collection (QHAPDC), ***** Queensland Health Statistical Services Branch

TRIAL MANAGEMENT

The principal investigator (MRN) will oversee the conduct and progress of the trial. The principal investigator will screen the daily admission to ICU lists and liaise with the treating medical teams to optimise participant enrolment. No interim analyses are planned. The principal investigator will ensure all research personnel are appropriately orientated and trained, oversee recruitment and report to a trial safety monitoring committee who will monitor the progress and conduct of the trial. The trial safety monitoring committee will include: a physiotherapist and researcher experienced in the safe conduct of clinical trials with physiotherapy interventions; the trial coordinator and principal investigator; a critical care nurse who is also experienced in the safe conduct of clinical trials in critical care settings and two ICU medical consultants experienced with the safe conduct of clinical trials in intensive care units. One of the ICU medical consultants is employed externally to the study site. The principal investigator will provide an update report to the Safety Monitoring Committee on a monthly basis (and additional ad-hoc reports if an adverse event occurs). Additionally any Serious Adverse Events will be reported to the approving Metro South Human Research Ethics Committee that is overseeing the study. During in-bed cycling sessions, participants will be monitored for adverse events that will be recorded on the session data collection form. Adverse events that are being monitored for are; line or airway dislodgement, increase in ventatory support that persist greater than five minutes post exericse (e.g. increase PEEP or FiO2), blood oxygen desaturation less than 88% for more than one minute, increase in vasoactive or pain relief medication greater than 5mcg/min, increase in systolic blood pressure greater than 180mmHg for more than two minutes, increase in heart rate greater than 140bpm for more than two minutes, decrease in mean arterial

For peer review only

blood pressure less than 60mmHg for greater than two minutes and decrease in heart rate less than 50bpm for more than two minutes.

DISSEMINATION

Study results will be disseminated via publication in peer-reviewed literature and scientific conference presentations. It is anticipated that media releases in lay form will be completed to target the general community. Study results will also be placed on a university website for viewing by participants and other interested parties. There are no publication restrictions. Authorship eligibility guidelines as outlined in The Australian Code for the Responsible Conduct of Research [28] and consistent with those proposed by the International Committee of Medical Journal Editors will be followed to determine authorship [29].

DISCUSSION

Survival rates following critical illness are improving [30]; however, patients are experiencing deficits in physical and cognitive function that do not equal age matched peers 5 years after an episode of critical illness [31]. The delayed initiation of rehabilitaitve exercise interventions with critically ill patients may explain the limited effectiveness of clinical trials that have studied the effect of exercise interventions on patients functional outcomes [11]. A binational clinical trial that aimed to commence exercise interventions as early as possible with mechanically ventilated patients reported that despite the presence of a dedicated early mobility team, patient mobilisation out of bed while mechanical ventilation was in-situ was rare [32]. These results are substantiated by point prevalance studies from Australia, New Zealand and Germany that report that in 1281 patient days only one patient with an endotracheal tube was mobilised out-of-bed [3 33]. The presence of an endotracheal tube is negatively correlated with out-of-bed mobilisation in the United States with a reported odds ratio of 0.1, [95% CI, 0.05-0.2] [34]. Studies have reported that following a period of critical illness, rehabilitative interventions do not hasten recovery when they are provided post acute hospital discharge [15 35]. Consequently this trial will provide valuable clinical trial evidence regarding the effect of an exercise intervention initiated early in the critically ill patient's illness. Specifically, it will report empirical data about the effect of the intervention on the rate of skeletal muscle wasting, and whether early exercise interventions that can be feasibly implemented among people who are mechanically ventilated are associated with improved physical, cogntive and health related quality of life outcomes.

CYCLIST is a Phase IIb randomised controlled trial that is powered to investigate if the early application of an additional in-bed cycling intervention is able to reduce the rate of skeletal muscle

For peer review only

atrophy of patients' quadriceps muscle during and immediately following a period of critical illness, in comparison to usual care. Secondary outcomes evaluated in this study will assist sample size calculations for future studies to assess for efficacy of functional outcomes. The study will also aid planning of future rehabilitation based clinical trials with regard to rate of participant recruitment. The investigators are planning to meet at the completion of the study to facilitate reflection on aspects of the study that could be improved and to consider whether a definitive Phase III RCT is warranted. This will include consideration of the evidence of effect on primary and secondary outcomes, participant recruitment and adherence to the in-bed cycling protocol and rate, as well as acceptability of the intervention.

The strengths of this study are the implementation of the in-bed cycling intervention as soon as feasible (including while patients are still mechanically ventilated), with clear commencement and stopping rules. Another strength of the study is the blinded assessment of muscle structure, strength and function assessments. Measurement of the effect of early in-bed cycling on quality of life post hospital discharge is another strength that will assist to demonstrate if potential gains made early during a period of critical illness correspond to lasting functional improvements. It is expected that this study will also provide insights regarding the feasibilty of the in-bed cycling intervention with critically ill patients from the rates of compliance and completion of the in-bed cycling exercise intervention. The acceptability of in-bed cycling intervention from the perspective of critically ill patients' will be sought through a questionnaire and provide new information to inform potential implementation strategies for this intervention. A potential limitation of the study may be difficulty completing functional assessment measures at ICU discharge and at one week post-ICU discharge with patients who are either profoundly weak or present with an acute cognitive dysfunction. Further, usual care physiotherapy interventions will be delivered by treating teams independent of the study and prioritised over in-bed cycling. The frequency, intensity, type and duration of all usual care physiotherapy interventions are not being prospectively recorded as part of the trial outcomes. As the study design is a randomised controlled trial it is anticipated that the usual care physiotherapy will be similar across groups. Also this study is being conducted at a single centre and therefore results may need to be interpreted will caution.

Word Count: 3875

For peer review only

Contributors

MRN contributed to study conception, design, grant acquisition, trial management (including intervention coordination, data collection), data management, protocol drafting, appraisal and editing. SMM, LMA and JW contributed to study conception, design, grant acquisition, analysis plan, data management, protocol drafting, appraisal and editing. AGB contributed to study analysis plan, grant acquisition, data management, protocol drafting, appraisal and editing. All authors read and approved the final manuscript.

Funding

This is an investigator initiated trial without external sponsors. Following a competitive peer review process Metro South Health Study, Education and Research Trust Account (SERTA) awarded this study a grant in 2015. MRN has also been awarded a competitive Princess Alexandra Research Support Scheme Postgraduate Scholarship to conduct this study. SMM (#1090440) and AGB (#1117784) are supported by National Health and Medical Research Council (NHMRC) fellowships. In addition, this study is receiving in-kind support in the form of personnel and administrative support from Metro South Health (Queensland) to enable the study to be conducted. No funding body had a role in study design, collection, management, analysis, and interpretation of data; writing of the report; and the decision to submit the report for publication.

Competing interests

None declared

Access to data

The principal investigator MRN will have full access to the final trial dataset. Associate investigators SMM, LMA, JW and AGB will have access to the final de-identified trial dataset. Other investigators will be granted access to sections of the final trial dataset. Dataset sections may include de-identified demographic data and information that pertains to data collected by the professional discipline of the associate investigator.

Data Sharing Statement

No additional data are available, though details on statistical analysis are available from the corresponding author on request.

Ancillary and post-trial care

Given the low risk of adverse events no specific provisions for ancillary and post-trial care have been made. If patients from either allocated group require follow-up services appropriate referrals for follow-up care would be made.

Ethics approval

Research Ethics Approval

Human research ethics approvals for this study have been gained from Metro South Human Research Ethics Committee (EC00167) on the 28th April 2016 (HREC/16/QPAC/193), and subsequent approval following an administrative review from Queensland University of Technology Human Research Ethics Committee (QUT reference number: 1600000441). Ethics approval has been granted until 28th April 2019.

Site specific approval (SSA) has been granted by Metro South Centres for Health Research, Research Governance (SSA/16/QPAH/195) on the 1st June 2016.

CYCLIST Study Protocol Version 2.1 dated 30th March 2017 was approved on 13th April 2017.

Participant recruitment commenced on 26th July 2016.

Provenance and peer review

Not commissioned; externally peer reviewed.

For peer review only

References:

1. Puthucheary ZA, Rawal J, McPhail M, et al. Acute Skeletal Muscle Wasting in Critical Illness. *JAMA* 2013;310(15):1591-600.

2. Gosselink R, Bott J, Johnson M, et al. Physiotherapy for adult patients with critical illness: recommendations of the European Respiratory Society and European Society of Intensive Care Medicine Task Force on Physiotherapy for Critically Ill Patients. *Intensive Care Med* 2008;34(7):1188-99.

3. Berney SC, Harrold M, Webb SA, et al. Intensive care unit mobility practices in Australia and New Zealand: a point prevalence study. *Crit Care Resusc* 2013;15(4):260-5.

4. Fan E. Critical illness neuromyopathy and the role of physical therapy and rehabilitation in critically ill patients. *Respir Care* 2012;57(6):933-44; discussion 44-6.

5. Kho ME, Martin RA, Toonstra AL, et al. Feasibility and safety of in-bed cycling for physical rehabilitation in the intensive care unit. *Journal of critical care* 2015;30(6):1419.e1-19.e5.

6. Kho ME, Molloy AJ, Clarke FJ, et al. TryCYCLE: A Prospective Study of the Safety and Feasibility of Early In-Bed Cycling in Mechanically Ventilated Patients. *PloS one* 2016;11(12):e0167561.

7. Pires-Neto CR, Fogaça Kawaguchi YM, Sayuri Hirota A, et al. Very Early Passive Cycling Exercise in Mechanically Ventilated Critically Ill Patients: Physiological and Safety Aspects - A Case Series. *PloS one* 2013;8(9):e74182.

8. Burtin C, Clerckx B, Robbeets C, et al. Early exercise in critically ill patients enhances short-term functional recovery. *Crit Care Med* 2009;37(9):2499-505.

9. Parry SM, Berney S, Warrillow S, et al. Functional electrical stimulation with cycling in the critically ill: A pilot case-matched control study. *Journal of critical care* 2014;29(4):695.e1-e7.

10. Moss M, Nordon-Craft A, Malone D, et al. A randomized trial of an intensive physical therapy program for acute respiratory failure patients. *American journal of respiratory and critical care medicine* 2016;193.

11. Hodgson CL, Iwashyna TJ, Schweickert WD. All That Work and No Gain: What Should We Do to Restore Physical Function in Our Survivors? *American journal of respiratory and critical care medicine* 2016;193(10):1071.

12. Chan A-W, Tetzlaff JM, Gøtzsche PC, et al. SPIRIT 2013 explanation and elaboration: guidance for protocols of clinical trials. *BMJ (Clinical research ed)* 2013;346(jan08 15):e7586-e86.

13. Harris PA, Taylor R, Thielke R, et al. Research electronic data capture (REDCap)—A metadata-driven methodology and workflow process for providing translational research informatics support. *Journal of Biomedical Informatics* 2009;42(2):377-81.

14. Borg G. Psychophysical scaling with applications in physical work and the perception of exertion. *Scandinavian Journal of Work, Environment & Health* 1990(1):55-58.

For peer review only

15. Berney S, Haines K, Skinner EH, et al. Safety and feasibility of an exercise prescription approach to rehabilitation across the continuum of care for survivors of critical illness. *Phys Ther* 2012;92(12):1524-35.

16. Tillquist M, Kutsogiannis DJ, Wischmeyer PE, et al. Bedside Ultrasound Is a Practical and Reliable Measurement Tool for Assessing Quadriceps Muscle Layer Thickness. *Journal of Parenteral and Enteral Nutrition* 2014;38(7):886-90.

17. De Jonghe B, Sharshar T, Lefaucheur JP, et al. Paresis acquired in the intensive care unit: a prospective multicenter study. *JAMA* 2002;288(22):2859-67.

18. Parry SM, Berney S, Granger CL, et al. A new two-tier strength assessment approach to the diagnosis of weakness in intensive care: an observational study. *Crit Care* 2015;19:52.

19. Hodgson C, Needham D, Haines K, et al. Feasibility and inter-rater reliability of the ICU Mobility Scale. *Heart Lung* 2014;43(1):19-24.

20. Huang M, Chan KS, Zanni JM, et al. Functional Status Score for the ICU: An International Clinimetric Analysis of Validity, Responsiveness, and Minimal Important Difference. *Critical Care Medicine* 2016:1.

21. Ragavan VK, Greenwood KC, Bibi K. The Functional Status Score for the Intensive Care Unit Scale: Is It Reliable in the Intensive Care Unit? Can It Be Used to Determine Discharge Placement? *Journal of Acute Care Physical Therapy* 2016;7(3):93-100.

22. ATS statement: guidelines for the six-minute walk test. *American journal of respiratory and critical care medicine* 2002;166(1):111-7.

23. Ely EW, Margolin R, Francis J, et al. Evaluation of delirium in critically ill patients: validation of the Confusion Assessment Method for the Intensive Care Unit (CAM-ICU). *Crit Care Med* 2001;29(7):1370-9.

24. Herdman M, Gudex C, Lloyd A, et al. Development and preliminary testing of the new five-level version of EQ-5D (EQ-5D-5L). *Quality of Life Research* 2011;20(10):1727-36.

25. Knaus WA, Wagner DP, Draper EA, et al. The APACHE III prognostic system. Risk prediction of hospital mortality for critically ill hospitalized adults. *Chest* 1991;100(6):1619-36.

26. Vincent JL, Moreno R, Takala J, et al. The SOFA (Sepsis-related Organ Failure Assessment) score to describe organ dysfunction/failure. On behalf of the Working Group on Sepsis-Related Problems of the European Society of Intensive Care Medicine. *Intensive Care Med* 1996;22(7):707-10.

27. Lepage B, Lamy S, Dedieu D, et al. Estimating the Causal Effect of an Exposure on Change from Baseline Using Directed Acyclic Graphs and Path Analysis. *Epidemiology* 2015;26(1):122-29.

28. Australian Code for the Responsible Conduct of Research. *Australian Government* 2007:1-41.

29. Editors. ICoMJ. Recommendations for the Conduct, Reporting, Editing and Publication of Scholarly Work in Medical Journals Available from: http://www.ICMJE.org. [accessed 01/27/2017.

For peer review only

30. Iwashyna TJ, Cooke CR, Wunsch H, et al. Population Burden of Long - Term Survivorship After Severe Sepsis in Older Americans. *Journal of the American Geriatrics Society* 2012;60(6):1070-77.

31. Herridge MS, Tansey CM, Matté A, et al. Functional disability 5 years after acute respiratory distress syndrome. *N Engl J Med* 2011;364.

32. Hodgson C, Bellomo R, Berney S, et al. Early mobilization and recovery in mechanically ventilated patients in the ICU: a bi-national, multi-centre, prospective cohort study. *Crit Care* 2015;19:81.

33. Nydahl P, Ruhl AP, Bartoszek G, et al. Early mobilization of mechanically ventilated patients: a 1-day point-prevalence study in Germany. *Crit Care Med* 2014;42.

34. Jolley SE, Moss M, Needham DM, et al. Point Prevalence Study of Mobilization Practices for Acute Respiratory Failure Patients in the United States. *Crit Care Med* 2017;45(2):205-15.

35. Elliott D, McKinley S, Alison J, et al. Health-related quality of life and physical recovery after a critical illness: a multi-centre randomised controlled trial of a home-based physical rehabilitation program. *Crit Care* 2011;15(3):R142.

For peer review only

Figure 1

Consort diagram giving flow of participants throughout the study. Abbreviations: n, number; ICU, intensive care unit.

For peer review only

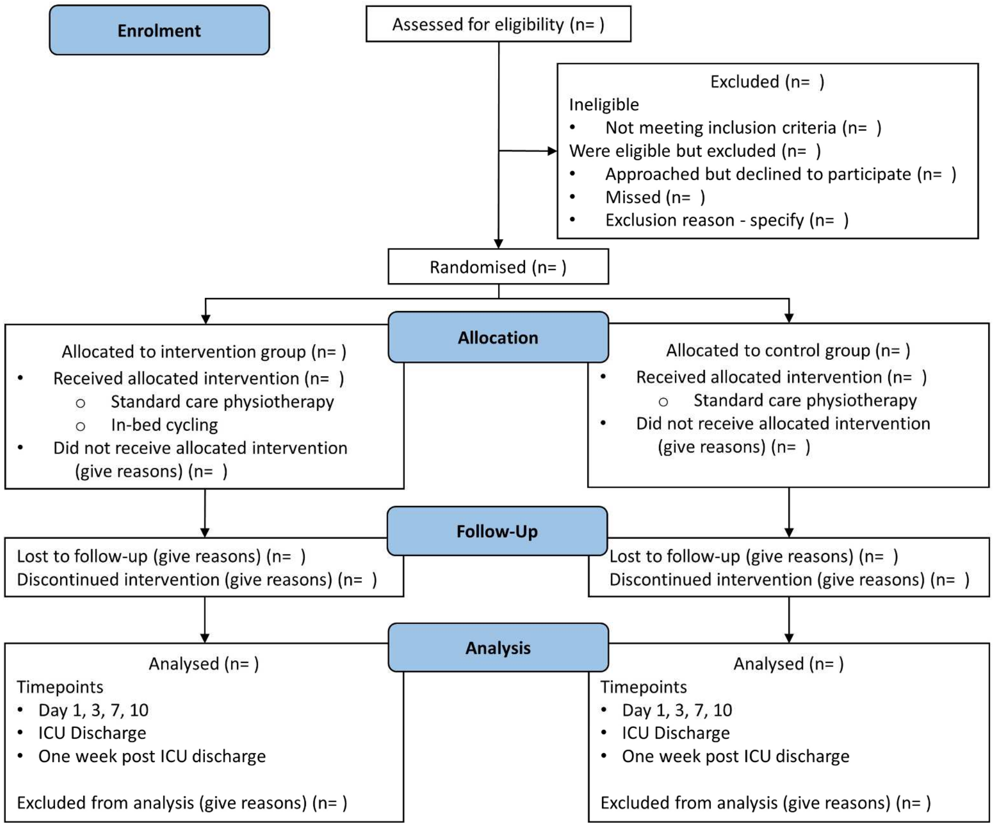

Figure 1

219x182mm (300 x 300 DPI)

Substitute Decision Maker Information Sheet

CYCLIST

Project Title	**Critical Care In-bed CYCLIng Study: A Preliminarily Randomised Controlled Trial: CYCLIST**
Site	Princess Alexandra Hospital
Principal investigator	Mr. Marc Nickels
Contact Person	Mr. Marc Nickels (07) 3176 2401,<br>or Mrs. Chelsea Davis (07) 3176-5523
Address	Physiotherapy Department, Princess Alexandra Hospital, Ipswich Road, Woolloongabba, QLD 4102
Phone Number	07 3176 2401 or (07) 3176-5523

Participant Information Sheet

Participation in this research is voluntary. Acting on behalf of the patient, we kindly ask that you read this information sheet about this clinical trial. If you are satisfied with the explanation and agree for the patient to participate in this trial, please provide your consent on the form provided. You should keep a copy of this sheet for your and for the future reference of the patient for who you are acting on behalf of.

Introduction

This study aims to investigate whether in-bed cycling in addition to standard care reduces the rate of thigh skeletal muscle wasting and is associated with improved functional and cognitive outcomes, in critically ill patients requiring more than 48 hours of mechanical ventilation. The study will also determine if patients who participate in in-bed cycling sessions are able to walk better after discharge from intensive care compared to people who receive standard care alone. This study will also investigate which factors are linked to better outcomes.

Patients who participate in the study will be randomly allocated into either an intervention group that receives in-bed cycling sessions in addition to standard care, or into a control group that receives standard care.

Background to experiment

Current physiotherapy exercise interventions with patients in intensive care do not occur until a patient is reliably able to follow instructions. Research has shown that patients rapidly lose muscle mass whilst they are in ICU. Recent studies have also shown that cycling in-bed whilst in ICU is safe. Research has also shown that patients who complete in-bed cycling whilst in ICU have improved ability to walk at hospital discharge. Currently it is not known what the effect of in-bed cycling is on maintaining thigh muscle mass. Ultrasound is a safe way to assess patients muscle mass without any radiation exposure that occurs with other investigations such as x-rays and computerised tomography scans.

For peer review only - http://bmjopen.bmj.com/site/about/guidelines.xhtml

Description of Experiment - methods and demands

This study will test if in-bed cycling in addition to standard care affects thigh muscle loss and if there is any difference in patients' time to walk and distance walked one week after intensive care discharge.

If you chose for the patient for whom you are the substitute decision maker for to participate this means:

a) The patient will be allocated, by a random (chance) selection process, to one of the following groups:
    - Standard care (control group): Physiotherapy exercise interventions that includes sitting on the edge of the bed, moving from a bed to a chair and walking.
    - Cycling group (in-bed cycling intervention group): at least five 30 minute in-bed cycling sessions in addition to 'standard care'. In-bed cycling sessions will continue until the patient is discharged from ICU and has completed a minimum of 5 in-bed cycling sessions. The in-bed cycling sessions will continue in the acute hospital ward if the patient is discharged from ICU prior to completing 5 in-bed cycling sessions).

b) Sonographers will use ultrasound to measure the size of the patients' thigh muscles during their stay in intensive care and one week after they leave the intensive care unit.
c) Physiotherapists will measure the patients' arm, leg muscle and hand grip strength.
d) A clinical nurse will record any incidence of confusion whilst the patient is in ICU.
e) Information about the time taken for the patient to commence standing, sitting out of bed, and walking both without assistance will be recorded.
f) One week after discharge from intensive care a physiotherapist will measure how far the patient can walk in 6 minutes.
g) Information about the patient such as; gender and age, and the patients' medical condition including; diagnosis, hospital length of stay, nutritional status, that is recorded in your hospital record will be accessed by researchers to aid in result analysis.
h) You will be asked questions about your quality of life in hospital and after you return home from hospital. You may also be asked questions about your experiences with treatments you received in hospital.
i) Data collected during this study may be utilised for future research purposes following appropriate subsequent ethical review and approval.

Risk & Discomfort

Other research has established that in-bed cycling is a safe exercise for critically ill patients to participate in. It is possible that in-bed cycling may increase pain or discomfort. However, care will be taken to minimise any potential discomfort that may be experienced, and pain relief medication may be increased if deemed appropriate by the doctors. Patients will be able to request for the in-bed cycling session to be stopped.

Benefits

There may be some benefit but we don't know how much direct benefit there will be to patients participating in the study. It is possible that participation in this study will reduce patients' amount of thigh muscle wasting due to lack of use. This may correspond to a patients' improved ability to walk following a period of critical illness. We also think that participation will benefit patients, and hospitals in the future, and you and the patient may feel satisfaction at your contribution to improving health care through research.

Withdrawing from the Study

Participation is entirely voluntary and if you decide the patient will not participate in this study this will not affect the medical care or treatment of the patient (for whom you are substitute decision maker),

For peer review only - http://bmjopen.bmj.com/site/about/guidelines.xhtml

in any way. If you choose for the patient to participate, you are free to withdraw your consent and to discontinue participation of the patient at any time, by telling the research nurse. Choosing for the patient not to participate or withdrawing your consent for their participation will not affect the treatment of the patient (for whom you are the substitute decision maker) for in any way.

Confidentiality

Data collected during this study will be treated confidentially. The research nurse and assistants will store data about the patient using a unique research number. The information will be safely stored at the hospital and Queensland University of Technology. Combined patient results of this study will be published in scientific journals and presented at conferences. However, the patient will not be referred to by name and your personal identity will not be revealed in any publication or report, without specific prior approval. Research data may be accessed by auditors, the ethics committee or regulatory authorities. All research records will be confidentially destroyed 7 years after the study.
Data collected during this study may be utilised for future research purposes following appropriate subsequent ethical review and approval. If data is utilised for future research purposes, it will continue to be treated confidentially.

Contact

If you have any questions now, or at a later time, we hope and expect that you will ask us. Please contact any of the researchers named on this form by contacting *the hospital principal investigator or research nurse*, and we will be happy to answer your questions. Contact details are at the top of this form.

Metro South HHS Human Research Ethics Committee (HREC), (EC00167) and the Queensland University of Technology HREC (EC00171) have approved this study. Should you wish to discuss the study with someone not directly involved, in particular, any matters concerning policies, information about the conduct of the study or the rights of the participant, or if you wish to make a confidential complaint at any time, you may contact;

Coordinator of the Metro South HHS Human Research Ethics Committee,
Translation Research Institute, Level 7,
Woolloongabba QLD 4102
Telephone (07) 3443-8049,
email: Ethicsresearch.pah@health.qld.gov.au

For peer review only - http://bmjopen.bmj.com/site/about/guidelines.xhtml

Queensland Government

Queensland University of Technology

Participant Consent Form

HREC No:	HREC/16/QPAH/193
Project Title:	Critical Care In-bed CYCLIng STudy Preliminary Randomised Controlled Trial: CYCLIST
Name of Researchers:	Mr Marc Nickels, Senior Physiotherapist, Princess Alexandra Hospital, Tel: (07) 3176-2401; Email: marc.nickels@health.qld.gov.au
	Dr Steven McPhail, Princial Research Fellow, Centre for Functioning and Health Research, Tel: (07) 3406 2266; Email: steven.mcphail@health.qld.gov.au

Thank you for agreeing for the patient for whom you are a substitute decision maker to participate in this important research study. Although you or they may not benefit personally, you will help provide valuable information to help us to deliver safe and effective care.

I have had the contents of this information sheet explained to me and I have been provided with a copy. I agree for the patient for whom I am a substitute decision maker for to be enrolled in the project and understand that they will be randomly allocated to either a usual care group or to a usual care plus in-bed cycling group.

Please read the following carefully, and sign below if you agree with these statements and are happy to for the patient for whom you are acting as a substitute decision maker for to participate in the study:

1. I have read and understood the information sheet and this consent form.
2. I have had the opportunity to ask questions about the study and these have been answered to my satisfaction.
3. I understand that this project is for research and that participation may not have a direct benefit to the participant.
4. I have been informed that the information collected about the participant in this study will remain confidential and will be adequately safeguarded, and that when results are published, they will be presented in such a way that individuals cannot be identified.
5. I understand that if I do agree for the patient to participate, I and they are free to withdraw our consent and to discontinue participation at any time without comment, and with no effect on their treatment or relations with the Hospital in any way, but that I do need to tell the research staff if I wish to withdraw the patient for whom I am a substitute decision maker for.
6. If I have any questions or comments about the study at any time I am free to contact Mr Marc Nickels on (07) 3176-2401 or the research nurse on (07) 3176 5523.
7. If I have any complaints about the ethical conduct of the study, I may direct these to the, Coordinator of the Ethics Committee, Princess Alexandra Hospital, on (07) 3176-8049.

For peer review only - http://bmjopen.bmj.com/site/about/guidelines.xhtml

I agree for the patient for whom I am a substitute decision maker for, to participate in the study and I give permission for authorised study personnel to extract details that pertain to this study from the patients' hospital medical record.

Name: ……………………………………….Signature………………………….. Date: __/__/__

Witness: .... Signature………………………….. Date: __/__/__

Enrolled by: .... Signature………………………….. Date: __/__/__

For peer review only

For peer review only - http://bmjopen.bmj.com/site/about/guidelines.xhtml

Queensland Government

Queensland University of Technology

Revocation of Consent Form - Participant

HREC No:	HREC/16/QPAH/193
Project Title:	Critical Care In-bed CYCLIng STudy Preliminary Randomised Controlled Trial: CYCLIST
Name of Researchers:	Mr Marc Nickels, Senior Physiotherapist,<br>Princess Alexandra Hospital, Tel: (07) 3176-2401; Email: marc.nickels@health.qld.gov.au
	Dr Steven McPhail, Princial Research Fellow, Centre for Functioning and Health Research, Tel: (07) 3406 2266; Email: steven.mcphail@health.qld.gov.au

(TO BE USED FOR PARTICIPANTS WHO WISH TO WITHDRAW FROM THE PROJECT)

- I hereby wish to WITHDRAW my consent for the patient for whom I am a substitute decision maker for to participate in the research proposal described above.
- I understand that such withdrawal WILL NOT jeopardise any treatment of the patient for whom I am a substitute decision maker for or relationship with the Princess Alexandra Hospital or Queensland University of Technology.

Participant's Name (printed)

____

______________________________ ________________

Signature Date

Please send to:
Clinical Research Nurse
Intensive Care Unit
Princess Alexandra Hospital
199 Ipswich Rd
Woolloongabba QLD 4102

For peer review only - http://bmjopen.bmj.com/site/about/guidelines.xhtml

SPIRIT
STANDARD PROTOCOL ITEMS: RECOMMENDATIONS FOR INTERVENTIONAL TRIALS

SPIRIT 2013 Checklist: Recommended items to address in a clinical trial protocol and related documents*

Section/item	Item No	Description	Location in manuscript
**Administrative information**			
Title	1	Descriptive title identifying the study design, population, interventions, and, if applicable, trial acronym	Title, page 1
Trial registration	2a	Trial identifier and registry name. If not yet registered, name of intended registry	Abstract, page 2
	2b	All items from the World Health Organization Trial Registration Data Set	Throughout manuscript
Protocol version	3	Date and version identifier	Additional information, page 17
Funding	4	Sources and types of financial, material, and other support	Additional information, page 16
Roles and responsibilities	5a	Names, affiliations, and roles of protocol contributors	Additional information, page 16
	5b	Name and contact information for the trial sponsor	Additional information page 16
	5c	Role of study sponsor and funders, if any, in study design; collection, management, analysis, and interpretation of data; writing of the report; and the decision to submit the report for publication, including whether they will have ultimate authority over any of these activities	Additional information, page 16

For peer review only

	5d	Composition, roles, and responsibilities of the coordinating centre, steering committee, endpoint adjudication committee, data management team, and other individuals or groups overseeing the trial, if applicable (see Item 21a for data monitoring committee)	Trial Management, page 13
**Introduction**			
Background and rationale	6a	Description of research question and justification for undertaking the trial, including summary of relevant studies (published and unpublished) examining benefits and harms for each intervention	Introduction, page 3
	6b	Explanation for choice of comparators	Introduction, page 3
Objectives	7	Specific objectives or hypotheses	Objectives, page 5
Trial design	8	Description of trial design including type of trial (eg, parallel group, crossover, factorial, single group), allocation ratio, and framework (eg, superiority, equivalence, noninferiority, exploratory)	Methods, Study Design and Setting, page 5
**Methods: Participants, interventions, and outcomes**			
Study setting	9	Description of study settings (eg, community clinic, academic hospital) and list of countries where data will be collected. Reference to where list of study sites can be obtained	Methods, Study Design and Setting, page 5
Eligibility criteria	10	Inclusion and exclusion criteria for participants. If applicable, eligibility criteria for study centres and individuals who will perform the interventions (eg, surgeons, psychotherapists)	Table 1, Inclusion and exclusion criteria, page 6
Interventions	11a	Interventions for each group with sufficient detail to allow replication, including how and when they will be administered	Study Intervention, page 7-8

For peer review only

	11b	Criteria for discontinuing or modifying allocated interventions for a given trial participant (eg, drug dose change in response to harms, participant request, or improving/worsening disease)	Table 2, Safety Guidelines, page 8
	11c	Strategies to improve adherence to intervention protocols, and any procedures for monitoring adherence (eg, drug tablet return, laboratory tests)	Study Intervention, page 8
	11d	Relevant concomitant care and interventions that are permitted or prohibited during the trial	Study Intervention, page 8
Outcomes	12	Primary, secondary, and other outcomes, including the specific measurement variable (eg, systolic blood pressure), analysis metric (eg, change from baseline, final value, time to event), method of aggregation (eg, median, proportion), and time point for each outcome. Explanation of the clinical relevance of chosen efficacy and harm outcomes is strongly recommended	Outcomes, page 9<br>Table 3
Participant timeline	13	Time schedule of enrolment, interventions (including any run-ins and washouts), assessments, and visits for participants. A schematic diagram is highly recommended (see Figure)	Figure 1
Sample size	14	Estimated number of participants needed to achieve study objectives and how it was determined, including clinical and statistical assumptions supporting any sample size calculations	Sample size, page 10
Recruitment	15	Strategies for achieving adequate participant enrolment to reach target sample size	Trial Management page 13.
**Methods: Assignment of interventions (for controlled trials)**			

Allocation:			
Sequence generation	16a	Method of generating the allocation sequence (eg, computer-generated random numbers), and list of any factors for stratification. To reduce predictability of a random sequence, details of any planned restriction (eg, blocking) should be provided in a separate document that is unavailable to those who enrol participants or assign interventions	Randomisation, page 6
Allocation concealment mechanism	16b	Mechanism of implementing the allocation sequence (eg, central telephone; sequentially numbered, opaque, sealed envelopes), describing any steps to conceal the sequence until interventions are assigned	Randomisation, page 6
Implementation	16c	Who will generate the allocation sequence, who will enrol participants, and who will assign participants to interventions	Randomisation, page 6
Blinding (masking)	17a	Who will be blinded after assignment to interventions (eg, trial participants, care providers, outcome assessors, data analysts), and how	Data collection, page 12
	17b	If blinded, circumstances under which unblinding is permissible, and procedure for revealing a participant's allocated intervention during the trial	Data collection, page 12
**Methods: Data collection, management, and analysis**			

Data collection methods	18a	Plans for assessment and collection of outcome, baseline, and other trial data, including any related processes to promote data quality (eg, duplicate measurements, training of assessors) and a description of study instruments (eg, questionnaires, laboratory tests) along with their reliability and validity, if known. Reference to where data collection forms can be found, if not in the protocol	Data collection, page 10-12
	18b	Plans to promote participant retention and complete follow-up, including list of any outcome data to be collected for participants who discontinue or deviate from intervention protocols	Study Intervention, page 8
Data management	19	Plans for data entry, coding, security, and storage, including any related processes to promote data quality (eg, double data entry; range checks for data values). Reference to where details of data management procedures can be found, if not in the protocol	Data collection, page 10-12
Statistical methods	20a	Statistical methods for analysing primary and secondary outcomes. Reference to where other details of the statistical analysis plan can be found, if not in the protocol	Analysis, page 12-13
	20b	Methods for any additional analyses (eg, subgroup and adjusted analyses)	Analysis, Table 5, page 13
	20c	Definition of analysis population relating to protocol non-adherence (eg, as randomised analysis), and any statistical methods to handle missing data (eg, multiple imputation)	Analysis, page 12-13
**Methods: Monitoring**			

Data monitoring	21a	Composition of data monitoring committee (DMC); summary of its role and reporting structure; statement of whether it is independent from the sponsor and competing interests; and reference to where further details about its charter can be found, if not in the protocol. Alternatively, an explanation of why a DMC is not needed	Contributors, page 16
	21b	Description of any interim analyses and stopping guidelines, including who will have access to these interim results and make the final decision to terminate the trial	Trial management, page 13
Harms	22	Plans for collecting, assessing, reporting, and managing solicited and spontaneously reported adverse events and other unintended effects of trial interventions or trial conduct	Trial management, page 13
Auditing	23	Frequency and procedures for auditing trial conduct, if any, and whether the process will be independent from investigators and the sponsor	Trial management, page 13
**Ethics and dissemination**			
Research ethics approval	24	Plans for seeking research ethics committee/institutional review board (REC/IRB) approval	Abstract, page 2
Protocol amendments	25	Plans for communicating important protocol modifications (eg, changes to eligibility criteria, outcomes, analyses) to relevant parties (eg, investigators, REC/IRBs, trial participants, trial registries, journals, regulators)	Analysis, page 12
Consent or assent	26a	Who will obtain informed consent or assent from potential trial participants or authorised surrogates, and how (see Item 32)	Consent, page 5-6

w only

	26b	Additional consent provisions for collection and use of participant data and biological specimens in ancillary studies, if applicable	Not applicable
Confidentiality	27	How personal information about potential and enrolled participants will be collected, shared, and maintained in order to protect confidentiality before, during, and after the trial	Data Collection, page 11
Declaration of interests	28	Financial and other competing interests for principal investigators for the overall trial and each study site	Additional information, page 16
Access to data	29	Statement of who will have access to the final trial dataset, and disclosure of contractual agreements that limit such access for investigators	Additional information, page 16
Ancillary and post-trial care	30	Provisions, if any, for ancillary and post-trial care, and for compensation to those who suffer harm from trial participation	Additional information, page 16
Dissemination policy	31a	Plans for investigators and sponsor to communicate trial results to participants, healthcare professionals, the public, and other relevant groups (eg, via publication, reporting in results databases, or other data sharing arrangements), including any publication restrictions	Dissemination, page 13
	31b	Authorship eligibility guidelines and any intended use of professional writers	Dissemination, page 13
	31c	Plans, if any, for granting public access to the full protocol, participant-level dataset, and statistical code	Dissemination, page 13
**Appendices**			

For peer review only

Informed consent materials	32	Model consent form and other related documentation given to participants and authorised surrogates	Substitute Decision Maker Consent Form
Biological specimens	33	Plans for collection, laboratory evaluation, and storage of biological specimens for genetic or molecular analysis in the current trial and for future use in ancillary studies, if applicable	Not applicable

*It is strongly recommended that this checklist be read in conjunction with the SPIRIT 2013 Explanation & Elaboration for important clarification on the items. Amendments to the protocol should be tracked and dated. The SPIRIT checklist is copyrighted by the SPIRIT Group under the Creative Commons "Attribution-NonCommercial-NoDerivs 3.0 Unported" license.

For peer review only
